# Multifractal Desynchronization of the Cardiac Excitable Cell Network During Atrial Fibrillation. I. Multifractal Analysis of Clinical Data

**DOI:** 10.3389/fphys.2017.01139

**Published:** 2018-03-26

**Authors:** Guillaume Attuel, Evgeniya Gerasimova-Chechkina, Francoise Argoul, Hussein Yahia, Alain Arneodo

**Affiliations:** ^1^Geometry and Statistics in Acquisition Data, Centre de Recherche INRIA, Talence, France; ^2^Laboratory of Physical Foundation of Strength, Institute of Continuous Media Mechanics UB RAS, Perm, Russia; ^3^Laboratoire Ondes et Matières d'Aquitaine, Université de Bordeaux, Centre National de la Recherche Scientifique, UMR 5798, Talence, France

**Keywords:** mechanisms of atrial fibrillation, heart electrical activity, multifractal analysis, wavelet transform modulus maxima method, two-point magnitude correlation analysis, multifractal noise

## Abstract

Atrial fibrillation (AF) is a cardiac arrhythmia characterized by rapid and irregular atrial electrical activity with a high clinical impact on stroke incidence. Best available therapeutic strategies combine pharmacological and surgical means. But when successful, they do not always prevent long-term relapses. Initial success becomes all the more tricky to achieve as the arrhythmia maintains itself and the pathology evolves into sustained or chronic AF. This raises the open crucial issue of deciphering the mechanisms that govern the onset of AF as well as its perpetuation. In this study, we develop a wavelet-based multi-scale strategy to analyze the electrical activity of human hearts recorded by catheter electrodes, positioned in the coronary sinus (CS), during episodes of AF. We compute the so-called multifractal spectra using two variants of the wavelet transform modulus maxima method, the moment (partition function) method and the magnitude cumulant method. Application of these methods to long time series recorded in a patient with chronic AF provides quantitative evidence of the multifractal intermittent nature of the electric energy of passing cardiac impulses at low frequencies, i.e., for times (≳0.5 s) longer than the mean interbeat (≃ 10^−1^ s). We also report the results of a two-point magnitude correlation analysis which infers the absence of a multiplicative time-scale structure underlying multifractal scaling. The electric energy dynamics looks like a “multifractal white noise” with quadratic (log-normal) multifractal spectra. These observations challenge concepts of functional reentrant circuits in mechanistic theories of AF, still leaving open the role of the autonomic nervous system (ANS). A transition is indeed observed in the computed multifractal spectra which group according to two distinct areas, consistently with the anatomical substrate binding to the CS, namely the left atrial posterior wall, and the ligament of Marshall which is innervated by the ANS. In a companion paper (II. Modeling), we propose a mathematical model of a denervated heart where the kinetics of gap junction conductance alone induces a desynchronization of the myocardial excitable cells, accounting for the multifractal spectra found experimentally in the left atrial posterior wall area.

## 1. Introduction

AF is an arrhythmia originating in the rapid and irregular electrical activity of the atria (the heart's two upper chambers) that causes their pump function to fail, increasing up to fivefold the risk of embolic stroke (Wolf et al., 1978, 1991; Attuel et al., 1986; Lip and Lane, 2015). Isolated short episodes of tachy-arrhythmias may be normal, but if they become more frequent and last longer than 1 min then paroxysmal AF is declared. This condition alone requires treatment of the atrial electrophysiological substrate, all the more so as AF often coexists with and predisposes to heart failure, with increased morbidity and mortality (Middlekauff et al., 1991; Stevenson and Stevenson, 1999; Wang et al., 2003). Management of AF by drug therapy aims at controlling either the ventricular rate, for instance by use of negative chronotropes such as beta-adrenergic blockers, or the rhythm, by use of anti-arrhythmic drugs interfering with ionic membrane currents of the excitable cells to prolong the action potential duration (APD), in combination with anticoagulants, but it does not lead to a cure (Roy et al., 2008; Al-Khatib et al., 2014). Since the work of Haïssaguerre et al. (1998), radio-frequency ablation of the pulmonary veins in the left atria has been developed for patients with paroxysmal AF as drug efficacy was found to be poor or even to become pro-arrhythmic (Echt et al., 1991; The CAST II investigators, 1992). This type of intervention seeks to punctually destroy sources of abnormal focal electrical activity susceptible to trigger the arrhythmia. Alternative strategies that have been developed lately include compartmenting the atrium in order to block possible reentrant circuits, or even directly targeting areas of abnormally fractionated activity (Nademanee et al., 2004; Camm et al., 2010). Despite the association of various strategies, clinical efficiency remains disappointing (Ganesan et al., 2013; Takigawa et al., 2014). The rate of AF recurrences after an initial ablation procedure treating paroxysmal AF increases with time (Bertaglia et al., 2010), necessitating multiple redos, and most patients suffering persistent AF are resistant to treatment (Camm et al., 2010; Verma et al., 2015; Wynn et al., 2016).

The prevailing electrophysiological concepts describing tachy-arrhythmias are more than a century old. They involve abnormal automaticity and conduction (Janse, 1997). Initiation and maintenance are thought to arise from a vulnerable substrate prone to the emergence of multiple self-perpetuating reentry circuits, also called “multiple wavelets” (Moe and Abildskov, 1959; Moe et al., 1964). Reentries may be driven structurally, for instance because of locally high fibrous tissue content which badly conducts, or functionally because of high spatial dispersion of decreased refractoriness and APD (Misier et al., 1992). The latter is coined the leading circle concept with the clinically more relevant notion of a critical “wavelength” (in fact the length) of the cardiac impulse (Allessie et al., 1977; Smeets et al., 1986; Rensma et al., 1988; Attuel et al., 1989). The related concept of vulnerability was originally introduced to uncover a physiological substrate evolving from normality to pathology. It was found in vulnerable patients that high rate frequency would invariably lead to functional disorder as cardiac cells would no longer properly adapt their refractoriness (Attuel et al., 1982). Mathematical models have managed to exhibit likewise phenomena, with the generation of breaking spiral waves in various conditions (Ito and Glass, 1991; Karma, 1993). The triggering role of abnormal ectopic activity of the pulmonary veins has been demonstrated on patients with paroxysmal AF resistant to drug therapy (Haïssaguerre et al., 1998), but its origin still remains poorly understood. This region is highly innervated with sympathetic and parasympathetic stimulation from the ANS (Tan et al., 2007; Ulphani et al., 2007; Arora, 2012). In particular, Coumel et al. (Coumel et al., 1978; Coumel, 1994) have revealed the pathophysiological role of the vagal tone on a vulnerable substrate. It is frequently observed that rapid tachycardia of ectopic origin transits to AF. This is known to result from electrical remodeling. As described for the first time by Allessie et al. (Allessie, 1998), remodeling is a transient and reversible process by which the impulse properties such as its refractory period are altered during the course of the arrhythmia, promoting its perpetuation: “AF begets AF” (Wijffels et al., 1995). Under substantial beating rate increase, cells may undergo remodeling to overcome the toxicity of their excessive intercellular calcium loading, by a rapid down regulation (a few minutes) of their L-type calcium membrane current (Yue et al., 1997; Nattel et al., 2008). Moreover, other ionic channel functions are also modified such as the potassium channel function, inducing a change in the conduction properties including the conduction velocity (Iwasaki et al., 2011; Nattel and Harada, 2014). The intercellular coupling at the gap junction level shows also alterations of their connexin expression and dispersion (Severs et al., 2008). For more details, one may consult van Marion et al. (2015) and Zipes et al. (2017).

In this study, we delve into the complexity of voltage signals recovered with bipolar electrodes in the CS during chronic AF. Attempts to assess visually the spatio-temporal complexity of voltage signals using maps of unipolar electrodes revealed various complex patterns of activity not all compatible with reentries (Konings et al., 1994, 1997). We use here two declinations of a wavelet-based multi-scale method, the moment (partition function) method and the magnitude cumulant method (Muzy et al., 1994; Arneodo et al., 2008), as originally introduced in the field of fully developed turbulence (Muzy et al., 1991). This methodology has been extensively applied in different domains of fundamental and applied sciences, including geophysics (Venugopal et al., 2006), econophysics (Muzy et al., 2001), biology (Arneodo et al., 2011) and medicine (Gerasimova et al., 2014; Gerasimova-Chechkina et al., 2016). In the context of cardiac physiology, this methodology was shown to be valuable in assessing congestive heart failure from the monitoring of sinus heart rate variability (Ivanov et al., 1999, 2001; Goldberger et al., 2002). The wavelet-based multifractal analysis of the electric energy of passing cardiac impulses during AF reported in the paper provides unprecedented experimental estimates of the multifractal spectra in different heart areas. The reported results show that the electric energy dynamics looks like a log-normal “multifractal white noise” with no underlying multiplicative time-scale structure. Long time series recordings, at various locations throughout the whole atria of many patients, are mandatory to obtain and analyze with the aim at identifying how many multifractal scalings exist. For instance, what is the effect on multifractal scaling of altered cell network topologies, especially when fibrosis is present? Or how is such scaling correlated to the disease temporal evolution, with or without drug therapy or surgical intervention, when the substrate is remodeled? In a companion paper (Attuel et al., submitted), we start tackling the problem at source, in the simplest (1D) cell network topology, by exploring the possibility that the substrate function is modulated by the kinetics of conduction. A simple reversible mechanism of short term remodeling under rapid pacing is demonstrated, by which ionic overload acts locally (dynamical feedback) on the kinetics of gap junction conductance. The whole process may propagate and pervade the myocardium via electronic currents. No influence of the ANS is included and no structural inhomogeneities are taken into account. Then the complete network of excitable cells becomes desynchronized, with induced dispersion of remodeled refractoriness and APD, and abnormal automaticity. Contrary to existing mathematical models based on circuit reentries, a spatio-temporal multifractal intermittent dynamics emerges similar to the one found in the CS next to the left atrial posterior wall area, opening a new avenue toward the understanding of AF mechanisms of perpetuation.

## 2. Methods of analysis

The wavelet transform (WT) is a mathematical microscope (Arneodo et al., 1988, 1995b, 2008; Muzy et al., 1991, 1994) that is well suited for the analysis of complex non-stationary time-series such as those found in genomics (Nicolay et al., 2007; Arneodo et al., 2011; Audit et al., 2013) and physiological systems (Ivanov et al., 1999, 2001; Goldberger et al., 2002; Ciuciu et al., 2012; Chudácek et al., 2014; Gerasimova et al., 2014; Richard et al., 2015). Thanks to its ability to be blind to non-stationary low-frequency (polynomial) trends in the analyzed signal *E*(*t*) (or *E*(*x*)), it has been early recognized as well adapted to reveal the hierarchy that governs the temporal (or spatial) distribution of singularities of multifractal signals including singular measures and functions (Arneodo et al., 1988; Holschneider, 1988; Jaffard, 1989; Muzy et al., 1991, 1994; Mallat and Hwang, 1992). It is therefore implemented in robust methods capturing the self-similar intricate fractal structures hidden in signals that exhibit a typical “1/f noise” scaling as seen in the Fourier spectral density (Mandelbrot, 1982, 1998; West and Shlesinger, 1988).

### 2.1. The wavelet transform microscope: a singularity scanner

The WT is a time-scale decomposition method which consists in expanding signals in terms of wavelets constructed from a single function, the “analyzing wavelet” ψ, by means of translations and dilations (Grossmann and Morlet, 1984; Daubechies, 1992; Meyer, 1992; Mallat, 1998). The WT of a real-valued function *E* is defined as:

(1) $$T_{\psi }[E](t_{0},a)=\frac{1}{a}\int_{-\infty }^{+\infty }E(t)\psi (\frac{t-t_{0}}{a})dt,$$

where *t*_0_ is a time parameter and *a* (> 0) a scale parameter (inverse of frequency). By choosing a wavelet ψ which has its first *n*_ψ_ moments null $\left[\int t^{m}\psi \left(t\right)dt=0,0\leq m<n_{\psi }\right]$, it can be proven that the behavior of *T*_ψ_[*E*](*t*_0_, *a*) as a function of the scale *a*, as *a* → 0^+^, characterizes the local behavior of *E*(*t*) (Arneodo et al., 1988, 1995b, 2008; Jaffard, 1989; Muzy et al., 1991, 1994; Mallat and Hwang, 1992):

(2) $$T_{\psi }[E](t_{0},a)\sim a^{h(t_{0})},\;a\to 0^{+},$$

provided *n*_ψ_ > *h*(*t*_0_), where *h*(*t*_0_) is the point-wise Hölder exponent that characterizes the maximum regularity of the signal *E* at point *t*_0_. If *n* < *h*(*t*_0_) ≤ *n* + 1, the (*n* − 1)th derivative of *E*(*t*) is regular and its *n*th derivative is singular at *t*_0_. Thus the larger *h*(*t*_0_), the smoother the function, the faster the power-law decrease of *T*_ψ_[*E*] when *a* → 0^+^. For *h*(*t*_0_) = 0, *E*(*t*) is discontinuous and bounded at *t*_0_ and the wavelet transform no longer depends on *a*. For discontinuous “noise” signals, *h*(*t*_0_) < 0 and *T*_ψ_[*E*](*t*_0_, *a*) increases when *a* → 0^+^. For instance, *h*(*t*_0_) = −1 corresponds to a delta distribution at *t* = *t*_0_, while if almost everywhere $h\left(t\right)=-\frac{1}{2}$, the single exponent $H=\frac{1}{2}$ is characteristic of a “white” noise (Muzy et al., 1994). To resolve all the cusp singularities present in a function, the analyzing wavelet must be chosen to have enough vanishing moments to resolve the singularities with Hölder exponent *h*_max_, namely *n*_ψ_ ≥ *h*_max_. Since *h*_max_ is not known a priori, the most appropriate way to correctly estimate all singularities is to analyze the given function with analyzing wavelets of increasing order *n*_ψ_ until a robust estimate of the so-called spectrum of singularities is obtained (Bacry et al., 1993; Muzy et al., 1994; Arneodo et al., 1995b) (see section 2.2). In the present study, we use the successive derivatives of a Gaussian function $g^{\left(N\right)}\left(t\right)=\frac{d^{N}}{dt^{N}}\left(e^{-t^{2}/2}\right)$ as analyzing wavelets with *n*_ψ_ = *N* (Muzy et al., 1994; Arneodo et al., 1995b) (Figure S1).

### 2.2. A wavelet-based canonical multifractal formalism: the wavelet transform modulus maxima method

The wavelet transform modulus maxima (WTMM) method (Muzy et al., 1991, 1994; Bacry et al., 1993; Arneodo et al., 1995b, 2008) was originally developed to generalize box-counting techniques (Arneodo et al., 1987) and to remedy the limitations of structure functions method (Muzy et al., 1993) in performing multifractal analysis of one-dimensional (1D) velocity signals in fully-developed turbulence. It has proved very efficient to estimate scaling exponents and multifractal spectra (Muzy et al., 1994; Audit et al., 2002; Arneodo et al., 2008). From the deep analogy that links the multifractal formalism to thermodynamics (Bohr and Tél, 1988; Arneodo et al., 1995b), the WTMM method provides a canonical description (Arneodo et al., 1995b) of the distribution of point-wise Hölder exponents for finite *a*. The WTMM method allows therefore some control and mastering of finite-size effects and statistical convergence issues. As *a* → 0^+^, the thermodynamic limit is reached formally guaranteeing an equivalence with micro-canonical approaches such as found in Turiel et al. (2008). To account for the possible presence of oscillating (chirps) (Arneodo et al., 1995a), a grand-canonical multifractal formalism has also been developed for signals involving cusp and oscillating singularities (Arneodo et al., 1997b). The canonical 1D WTMM method has been generalized in 2D for the multifractal analysis of rough surfaces (Arneodo et al., 2000, 2003; Decoster et al., 2000; Roux et al., 2000) and for the analysis of 3D scalar and vector fields (Kestener and Arneodo, 2003, 2004; Arneodo et al., 2008) with successful applications in astrophysics (Khalil et al., 2006; Kestener et al., 2010; McAteer et al., 2010), geophysics (Venugopal et al., 2006; Roux et al., 2009), surface science (Roland et al., 2009), image processing (Mallat, 1998; Arneodo et al., 2003, 2008; Antoine et al., 2008), cellular biology (Khalil et al., 2007; Snow et al., 2008; Goody et al., 2010; Grant et al., 2010; Martinez-Torres et al., 2014, 2015) and medicine (Kestener et al., 2001; Arneodo et al., 2003; Khalil et al., 2009; Batchelder et al., 2014; Gerasimova-Chechkina et al., 2016; Marin et al., 2017). Note that alternative approaches to the WTMM method have been developed using discrete wavelet bases, including the recent use of wavelet leaders (Jaffard et al., 2007; Wendt et al., 2007).

#### 2.2.1. The method of moments

In 1D, the WTMM method (Muzy et al., 1991, 1993, 1994; Bacry et al., 1993; Arneodo et al., 1995b) consists in computing the WT skeleton defined, at each fixed scale *a*, by the local maxima L(a) of the WT modulus |*T*_ψ_[*E*](*t, a*)|. These WTMM are disposed on curves connected across scales called maxima lines (see **Figure 2C**). Along these maxima lines *l*, Mallat and Hwang (1992) have shown that Equation (2) also applies for the WTMM that behave as $|T_{\psi }\left[E\right]\left(t,a\right)|∽a^{h\left(t\right)}$, where *h*(*t*) is the Hölder exponent characterizing the singularity of the signal *E* at time *t*. The canonical multifractal formalism (Muzy et al., 1994; Arneodo et al., 1995b) characterizes the relative contributions of each Hölder exponent value via the estimate of the singularity spectrum *D*(*h*) defined as the fractal (Hausdorff) dimension of the set of points *t* where *h*(*t*) = *h*. This spectrum can be obtained by investigating the scaling behavior of partition functions defined in terms of WTMM (and which correspond to the moments of the WTMM probability distribution function):

(3) $$Z(q,a)=\sum_{l\in L(a)}|T_{\psi }[E](t,a){|}^{q}\sim a^{\tau (q)},\;a\to 0^{+},$$

where *q* ∈ ℝ, and L(a) is the set of all maxima lines *l* that satisfy: l∈L(a), if ∀*a*′ ≤ *a*, ∃(*t, a*′) ∈ *l*. In the framework of the analogy with thermodynamics (Bohr and Tél, 1988; Arneodo et al., 1995b), *q* and τ(*q*) play respectively the role of an inverse temperature and a free energy. The main result of the canonical wavelet-based multifractal formalism is that in place of energy and entropy (i.e., the variables conjugated to *q* and τ), we have *h*, the Hölder exponent, and *D*(*h*), the singularity spectrum. This means that the singularity spectrum of *E*(*t*) is a convex function that can be calculated from the Legendre transform of the partition function scaling exponents τ(*q*) (Bacry et al., 1993; Muzy et al., 1993, 1994; Arneodo et al., 1995b):

(4) $$D(h)=\underset{q}{\min}[qh-\tau (q)].$$

In practice, to avoid instabilities in performing the Legendre transform, we instead compute the following expectation values (Muzy et al., 1994; Arneodo et al., 1995b), analogous to the fundamental thermodynamic relations, by inversion of Equation (4):

(5) $$h(q,a)=\frac{\partial }{\partial q}\ln(Z(q,a))=\sum_{l\in L(a)}\ln\left(\left|T_{\psi }[E](t,a)\right|\right)\cdot W_{\psi }[E](q,l,a),$$

and

(6) $$\begin{array}{l} D(q,a)=q\frac{\partial }{\partial q}\ln(Z(q,a))-\ln(Z(q,a))\\ \;=\sum_{l\in L(a)}W_{\psi }[E](q,l,a)\cdot \ln\left(W_{\psi }[E](q,l,a)\right), \end{array}$$

where $W_{\psi }\left[E\right]\left(q,l,a\right)=|T_{\psi }\left[E\right]\left(t,a\right){|}^{q}/Z\left(q,a\right)$ corresponds to the Bolzmann weight in the analogy that connects the multifractal formalism to thermodynamics (Arneodo et al., 1995b). Then, from the slopes of *h*(*q, a*) and *D*(*q, a*) vs. ln *a*, we get *h*(*q*) and *D*(*q*), and therefore the *D*(*h*) singularity spectrum as a curve parametrized by *q*. For further mathematical developments on the 1D WTMM method, we refer the reader to Bacry et al. (1993) and Jaffard (1997a,b).

#### 2.2.2. The method of magnitude cumulants

With the previous method of moments, to compute the entire τ(*q*) curve, we need to perform linear regression fits of ln *Z*(*q, a*) vs. ln *a* (Equation 3) for a wide range of *q* values and then to proceed to a polynomial fit of the τ(*q*) data prior to the Legendre transform (Equation 4) to get the *D*(*h*) singularity spectrum. An alternative method based on magnitude cumulants has been introduced by Delour et al. (2001) to minimize the number of linear regression fits (as few as 3) while still adequately inferring and accurately estimating the nonlinear behavior of the τ(*q*) spectrum. This method is based on the following reasoning. The computation of the partition function *Z*(*q, a*) amounts to computing the following arithmetic mean of the WTMM to the power *q*:

(7) $$\langle |T_{a}{|}^{q}\rangle =\frac{1}{N_{a}}Z(q,a),$$

where we simplified notations *T*_*a*_ ≡ *T*_ψ_[*E*](·, *a*), and where *N*_*a*_ is the number of maxima lines at scale *a*, which scales as $∽a^{-D_{f}}$, where *D*_*f*_ = −τ(0) is the fractal dimension of the support set of the singularities in the signal *E*(*t*). From Equations (3) and (7), we get the expansion

(8) $$\begin{array}{l} {}[\tau (q)+D_{f}]\ln a\sim \ln\{\langle e^{q\ln|T_{a}|}\rangle \},\\ \;\sim \sum_{n=1}^{\infty }C_{n}(a)\frac{q^{n}}{n!}, \end{array}$$

where *C*_*n*_(*a*) are the cumulants of the magnitude ln |*T*_*a*_|. Then from the behavior of these cumulants:

(9) $$\begin{array}{l} C_{1}(a)\equiv \langle \ln|T_{a}|\rangle \sim c_{1}\ln(a),\\ C_{2}(a)\equiv \langle \ln^{2}|T_{a}|\rangle -{\langle \ln|T_{a}|\rangle }^{2}\sim -c_{2}\ln a,\\ C_{3}(a)\equiv \langle \ln^{3}|T_{a}|\rangle -3\langle \ln^{2}|T_{a}|\rangle +2{\langle \ln|T_{a}|\rangle }^{3}\sim c_{3}\ln a, \end{array}$$

we get the following expansion formula for τ(*q*):

(10) $$\begin{array}{l} \tau (q)=-D_{f}\frac{q^{0}}{0!}+\sum_{n=1}^{\infty }\left[\frac{C_{n}(a)}{\ln a}\right]\frac{q^{n}}{n!},\\ \;=-c_{0}+c_{1}q-c_{2}q^{2}/2!+c_{3}q^{3}/3!\cdots \end{array}$$

where the coefficients *c*_*n*_ > 0 are estimated as the slope of *C*_*n*_(*a*) vs. ln (*n* = 1, 2, 3, , …), and *c*_0_ = *D*_*f*_.

The implication of the above developments is that we can estimate τ(*q*) from the polynomial expansion of Equation (10), where the coefficients are obtained from the log-log linear regressions of the cumulants of the magnitude *C*_*n*_(*a*) vs. ln(*a*) (Equation 9) (Delour et al., 2001). A quadratic log-normal τ(*q*) approximation would need only three such linear regressions, *c*_*n*_ = 0, ∀*n* > 2.

### 2.3. Monofractal vs. multifractal functions

Homogeneous monofractal signals (distributions) are signals with singularities of unique Hölder exponent *H*. Their τ(*q*) spectrum is a linear function of *q* with slope *c*_1_ = *H* (Equation 9). Monofractal scaling indeed means that the shape of the probability distribution function (pdf) of rescaled wavelet coefficients does not change across scales as expressed by the following relationship between the WTMM pdfs *P*_*a*_(*T*) and $P_{a^{\prime }}\left(T\right)$ at scale *a* and *a*′ > *a* respectively (Arneodo et al., 2002, 2011):

(11) $$P_{a}(T)={\left(\frac{a^{\prime }}{a}\right)}^{-H}P_{a^{\prime }}\left({\left(\frac{a^{\prime }}{a}\right)}^{-H}T\right).$$

A nonlinear τ(*q*) is the signature of multifractal signals with Hölder exponent *h*(*t*) fluctuating over time *t* (Muzy et al., 1991, 1994; Bacry et al., 1993; Arneodo et al., 1995b, 2002, 2008). In this study, we fit the τ(*q*) data by the so-called log-normal quadratic approximation $\tau \left(q\right)=-c_{0}+c_{1}q-c_{2}q^{2}/2$. The corresponding singularity spectrum has a quadratic single humped shape:

(12) $$D(h)=c_{0}-{\left(h-c_{1}\right)}^{2}/2c_{2},$$

where *c*_0_ = −τ(0) = *D*_*f*_ is the fractal dimension of the support of singularities of *E*(*t*), *c*_1_ is the value of *h* that maximizes *D*(*h*), and the intermittency coefficient *c*_2_ (Delour et al., 2001) characterizes the width of the *D*(*h*) spectrum as an indication of a change in WTMM coefficient statistics across scales. If *h*(*t*) fluctuates according to a pdf ρ(*h*), then (Castaing et al., 1990, 1993; Arneodo et al., 1997a, 1998c, 1999):

(13) $$P_{a}\left(T\right)=\int \rho (h){\left(\frac{a^{\prime }}{a}\right)}^{-h}P_{a^{\prime }}\left({\left(\frac{a^{\prime }}{a}\right)}^{-h}T\right)\;dh,$$

meaning that the pdf at scale *a* can be expressed as a weighted sum of dilated pdfs at larger scales *a* ′ >*a*. Let us point out that the monofractal situation (Equation 11) is recovered when assuming that ρ(*h*) = δ(*h* − *H*) in Equation (13).

Note that τ(2) = *c*_0_ + 2*c*_1_ − 2*c*_2_, also called the correlation dimension, is related to the “1/f” scaling exponent of the Fourier spectral density (Muzy et al., 1994; Mandelbrot, 1998):

(14) $$|\hat{E}(f){|}^{2}\sim f^{-\beta },\text{ with }\tau (2)=\beta -2.$$

### 2.4. In quest of an underlying time-scale multiplicative structure: the two-points magnitude correlation method

Multiplicative cascade processes (Arneodo et al., 1998b) are paradigmatic mechanisms generating multifractal distributions, with as historical examples the Kolmogorov–Obukhov log-normal energy cascade model of fully developed turbulence (Kolmogorov, 1962; Oboukhov, 1962; Mandelbrot, 1974), and the Multifractal Random Walk (MRW) model recently introduced to account for the intermittency observed in financial time series (Muzy et al., 2000; Bacry et al., 2001). But, if multifractal scaling implies some evolution of the WTMM statistics across scales, it does however not require any correlation of the wavelet coefficients across scales. In addition to the above one-point WTMM statistics, it is thus useful to study the two-point correlation function of the logs of the WTMM coefficients ln |*T*_*a*_(*t*)|, which determines the way the correlation structure of the Hölder exponents *h* (or singularities) changes with scale (Arneodo et al., 1998a,b). Defining the two-point magnitude correlation function *C*(*a*, Δ*t*) as:

(15) $$\begin{array}{l} C(a,\Delta t)=\langle \left(\ln|T_{a}(t)|-\langle \ln|T_{a}(t)|\rangle \right)\cdot (\ln|T_{a}(t+\Delta t)|\\ \;-\langle \ln|T_{a}(t)|\rangle )\rangle , \end{array}$$

and seeing how this correlation changes as a function of Δ*t* at scale *a*, provides information about the time-scale structure that underlies the multifractal properties of the considered signal. As demonstrated by Arneodo et al. (1998a,b) for random multiplicative cascades on wavelet dyadic trees (see also Meneveau and Sreenivasan, 1991):

(16) $$C(a,\Delta t)\sim -c_{2}\;\ln\Delta t,\;\Delta t>a,$$

where the proportionality coefficient *c*_2_ is the intermittency coefficient defined in Equation(9) (Note that *C*(*a*, Δ*t* = 0) ≡ *C*_2_(*a*) ∽ − *c*_2_ln *a*). Thus, by computing *C*(*a*, Δ*t*) from Equation (15) and plotting it as a function of ln Δ*t*, inferences can be made about long-range dependence and consistency with a multiplicative cascading process (Arneodo et al., 1998a,b). Applications of the two-point magnitude correlation method have already provided insight into a wide variety of problems, e.g., the validation of the log-normal cascade phenomenology of fully developed turbulence (Arneodo et al., 1998a,c, 1999) and of high resolution temporal rainfall (Venugopal et al., 2006; Roux et al., 2009), and the demonstration of the existence of a causal cascade of information from large to small scales in financial time series (Arneodo et al., 1998d; Muzy et al., 2001).

## 3. Description of data

### 3.1. Study design and population

The experimental data are hospital-based. We have analyzed data recorded in the atria of 8 patients with persistent or chronic AF, chosen without any prior explicit exclusion criteria. These patients were enrolled to undergo radio frequency ablation between 2010 and 2012, in the international cardiac electrophysiology service of public hospital CHU Haut-Lévêque in Pessac, France. All patients gave written informed consent to the investigation of data from the intervention. A protocol for clinic research was approved by the institutional Clinical Research and Ethics Committee. For this specific investigation of the data, the authors accessed fully anonymized and de-identified data. As representative of the results obtained with our set of patients, we report in this manuscript the results of a detailed wavelet-based multifractal analysis of five long time series specially recorded in one of the 8 patients with chronic AF.

### 3.2. Electric potential recording

**Figure 1:**
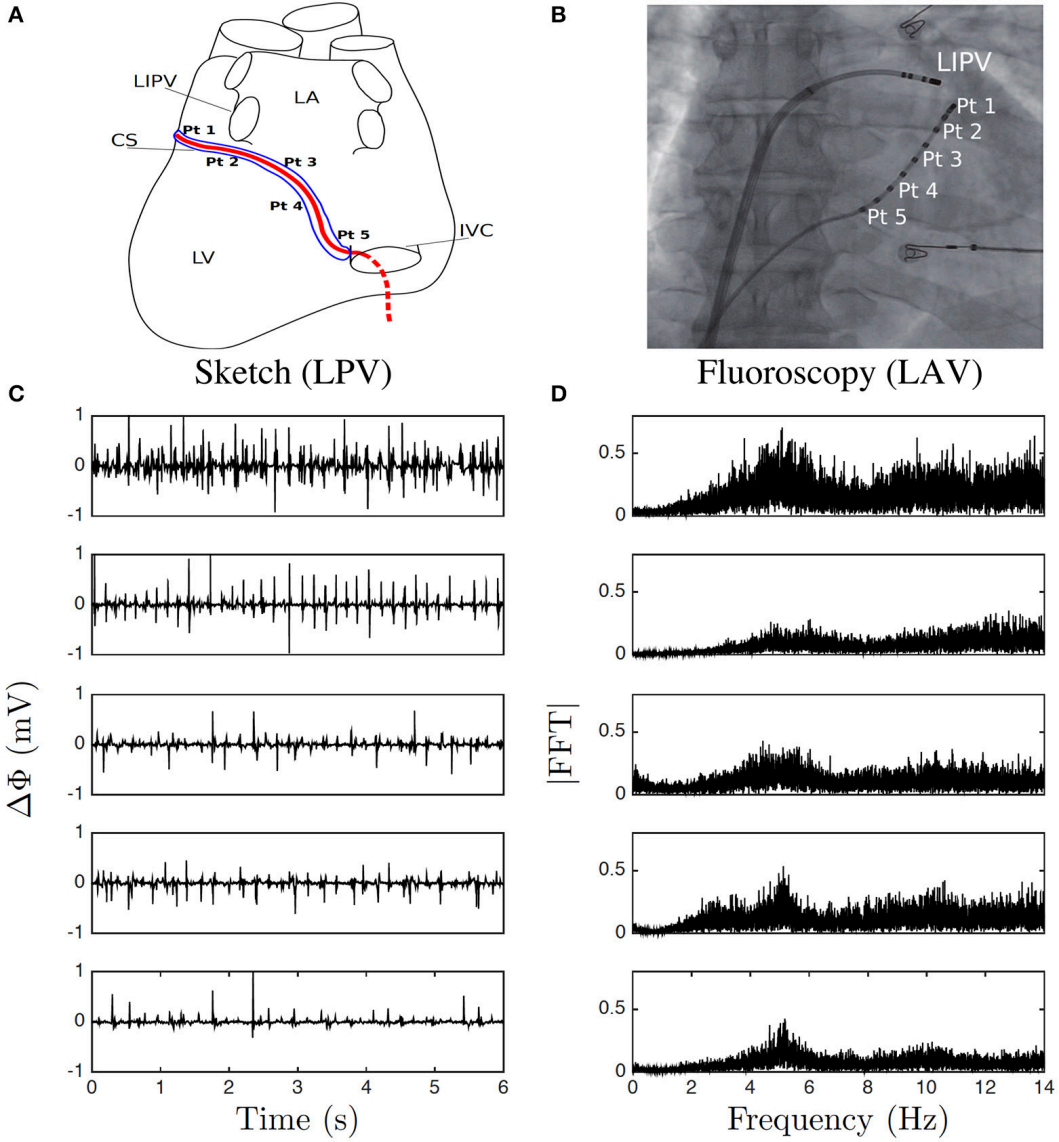
Electric potential recording. **(A)** Sketch of the positioning of the pairs of electrodes (Pt1–Pt5) along the catheter in the coronary sinus (CS); left posterior view (LPV) of the heart left ventricle (LV), left inferior pulmonary vein (LIPV), left atrium (LA), and inferior vena cava (IVC). **(B)** A Left anterior fluoroscopy view (LAV) of the atria showing the pairs of electrodes in the CS and one other catheter. **(C)** 6 s portions of Δϕ(*t*) recorded at the points Pt1, Pt2, Pt3, Pt4, and Pt5, from top to bottom. **(D)** Corresponding Fourier power spectra computed over the whole 422 s time series.

A steerable decapolar catheter, equipped with five 1 mm distant pairs of electrodes, each pair separated by 5 mm (Xtrem, Sorin Medical ©), was positioned in the CS as recommended in (Figures 1A,B). The distal leads of the catheter tip are positioned in a region near the left pulmonary veins, while the proximal leads lie closer to the right atrium orifice of the CS. This catheter was immobile and probed the electrical activity of those areas in the left atrium. Monitoring typically lasted the whole intervention which could take hours. Our file consists of 5 simultaneous recordings at points Pt1 to Pt5, from distal to proximal positioning along the vein (Figure 1A), each lasting 422 s with sampling time 10^−3^ s, a few minutes before the first ablation procedure started. The potential difference Δϕ(*t*) between each of the two electrodes in each pair was recorded, with the convention of distal minus proximal (Figure 1C). The normal to rapid frequency in sinus rhythm varies in the range 1Hz ≲ *f* ≲ 3 Hz, whereas during AF it is typically in the range 3Hz ≲ *f* ≲ 10Hz (Figure 1D). But the most obvious observation is that on-site recordings during AF contrast with the ones during sinus rhythm as the former seem to fluctuate randomly at even higher frequencies. Physiologically, a natural high frequency cut-off is somewhere in between 100Hz ≲ *f*_*c*_ ≲ 1, 000 Hz, which corresponds to the shortest characteristic time scale in a cardiac cell cycle, that is depolarization. Furthermore, AF is considered as the most irregular cardiac arrhythmia (Konings et al., 1994, 1997).

### 3.3. Local impulse energy

Each electrode averages the electric potential over its surface. During depolarization, ions flow through the cell membrane channels and the gap junction channels, inducing a rapid change of the electric potential (∽10^−2^s). Then, repolarization is a much smoother event. Bipolar electrodes are separated by a few millimeters which is typically the length scale of a depolarizing front in the atria with a conduction velocity *c* ∽ 10^−1^ m/s and a refractory period *RP* ∽ 10^−1^ s (Figures S2A,B), thus defining the so-called “wavelength” scale *c* × *RP* (Smeets et al., 1986; Rensma et al., 1988). Any spatio-temporal variation of the vulnerable substrate function happens over larger time scales. Thus the conduction velocity can be considered constant over such small scales and the bipolar electric potential difference is therefore “frozen.” It follows that the bipolar electric potential difference is locally advected with velocity *c*:

(17) $$\frac{\partial \Delta \phi (t)}{\partial t}=-c\vec{\nabla }\Delta \phi (t).$$

Under this assumption, the evaluation of the local electric energy of a cardiac impulse is straight-forward:

(18) $$\frac{\varepsilon }{2}{ℰ}^{2}(t)=\frac{\varepsilon }{2c^{2}}{\left(\frac{\partial }{\partial t}\Delta \phi (t)\right)}^{2},$$

where E is the electric field magnitude and ε is the dielectric bulk permitivity of the (inter-) cellular medium. Energy will thus peak when the impulse travels between the two electrodes. It fully incorporates ionic flux through membrane channels and electrotonic currents, specifically those taking place at the gap junctions. Because we have no means to assess the conduction velocity *c*, we will use in this study the following definition of the energy:

(19) $$E(t)={\left(\frac{\partial \Delta \phi (t)}{\partial t}\right)}^{2},$$

after dropping the term in front of the r.h.s. of Equation (18), i.e., ε/2*c*^2^ which remains constant at first order as long as the conduction velocity *c* does not fluctuate too much. To practically derive *E*(*t*) from the recorded Δϕ(*t*), we used an order 4 finite difference scheme on a oversampled ($\Delta t_{0}=10^{-4}$s) cubic-spline fitting of the data (Figures S2C,D). This is needed to estimate peaks in the energy within temporal windows as narrow as ∽10^−3^ s (Figures S2E,F). We have checked that the scaling properties displayed by *E*(*t*) in the low frequency range of interest here (0.08Hz ≲ *f* ≲ 2Hz) are not affected by this discretization scheme.

Let us note that Equation (19) is not without reminding the 1D surrogate dissipation approximation $E_{\text{dis}}\left(t\right)∽{\left(\partial \upsilon /\partial t\right)}^{2}$, where υ is the longitudial velocity, in fully developed (homogeneous and isotropic) turbulence under the Taylor hypothesis of “frozen” turbulence (Meneveau and Sreenivasan, 1991; Frisch, 1995).

### 3.4. Software and documentation

The numerical procedure to perform the WTMM analysis of 1D signals can be downloaded at

http://perso.ens-lyon.fr/benjamin.audit/LastWave

LastWave is open source software written in C. We recommend interested users to read the LastWave C-Application Programming Interface documentation and to contact the corresponding author to be directed to the part of the code of most relevance to them.

## 4. Results

### 4.1. One-point multifractal analysis of local impulse energy data

We first present an exhaustive step-by-step analysis of one of the impulse energy time-series recorded at the electrode Pt2 as an illustration of the intricacies involved in the methodology and as a demonstration that without a priori knowledge about the signal, a reliable multifractal analysis requires an iterative process between diagnosis and estimation until robustness is achieved. In Figure S3 is illustrated how the WT microscope is able to filter out the nonstationarities (polynomial trends) in *E*(*t*) (Figure S3A) when using analyzing wavelets *g*^(*N*)^ (Figure S1) of increasing order (Figures S3B–D).

#### 4.1.1. Multifractal analysis of the impulse energy data with the WTMM method of moments

**Figure 2:**
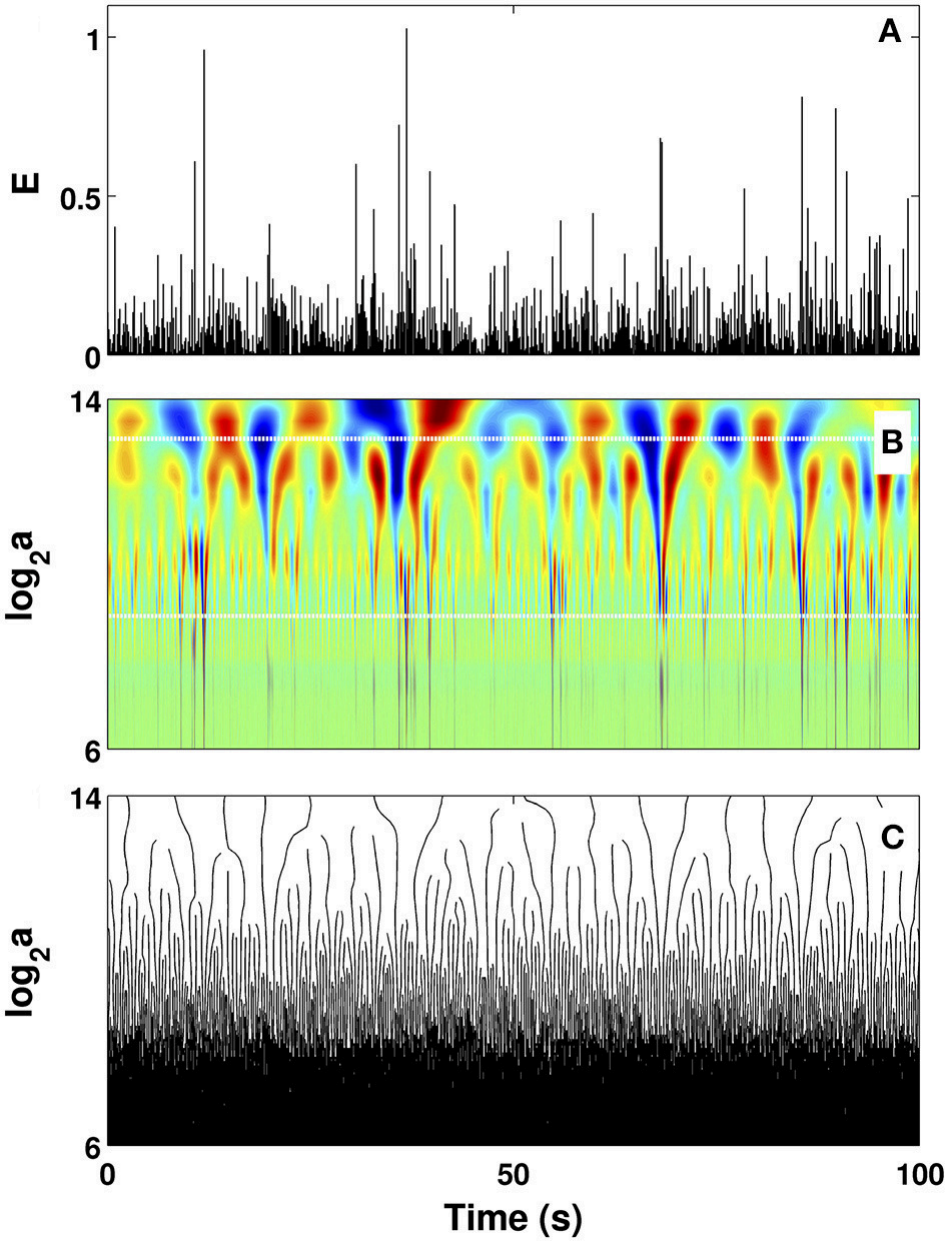
Wavelet transform of local impulse energy time-series. **(A)** A 100 s portion of *E*(*t*) (Equation 19) recorded at the electrode Pt2. **(B)** Time-scale WT representation of *E*(*t*) with the analyzing wavelet *g*^(3)^ (Figure S1). The modulus of the WT is coded, independently at each scale *a*, using 256 colors from black ($|T_{g^{\left(3\right)}}\left(t,a\right)|=0$) to red ($\underset{t}{\max}|T_{g^{\left(3\right)}}\left(t,a\right)|$). **(C)** WT skeleton defined by the maxima lines. The scale *a* = Δ*t*/Δ*t*_0_, where $\Delta t_{0}=10^{-4}$ s. In **(B)** the white horizontal dotted lines delimit the range of time scales (2^9^ ≤ *a* ≤ 2^13^) used to perform linear regression fit estimates of the τ(*q*) and *D*(*h*) multifractal spectra.

**Figure 3:**
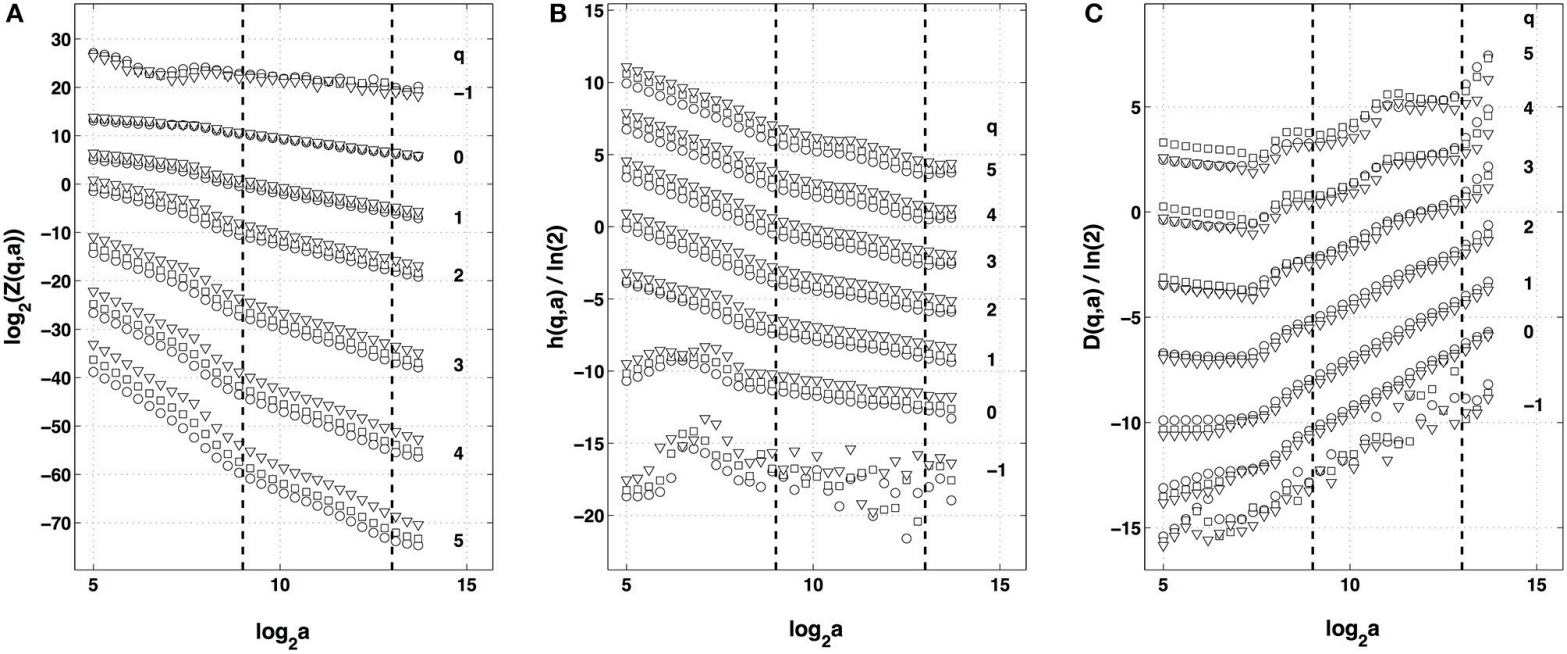
Multifractal analysis of local impulse energy time-series recorded at the electrode Pt2 with the WTMM method. **(A)** log_2_ *Z*(*q, a*) vs. log_2_ *a* (Equation 3). **(B)** *h*(*q, a*)/ln 2 vs. log_2_ *a* (Equation 5). **(C)** *D*(*q, a*)/ln 2 vs. log_2_ *a* (Equation 6). The computation were performed for different values from *q* = −1 to 5 with the analyzing wavelet *g*^(1)^ (▿), *g*^(2)^ (□) and *g*^(3)^ (◦) (Figure S1). The vertical dashed lines delimit the range of scales (2^9^ ≤ *a* ≤ 2^13^) used for the linear regression estimate of τ(*q*), *h*(*q*) and *D*(*q*) in Figure 4.

**Figure 4:**
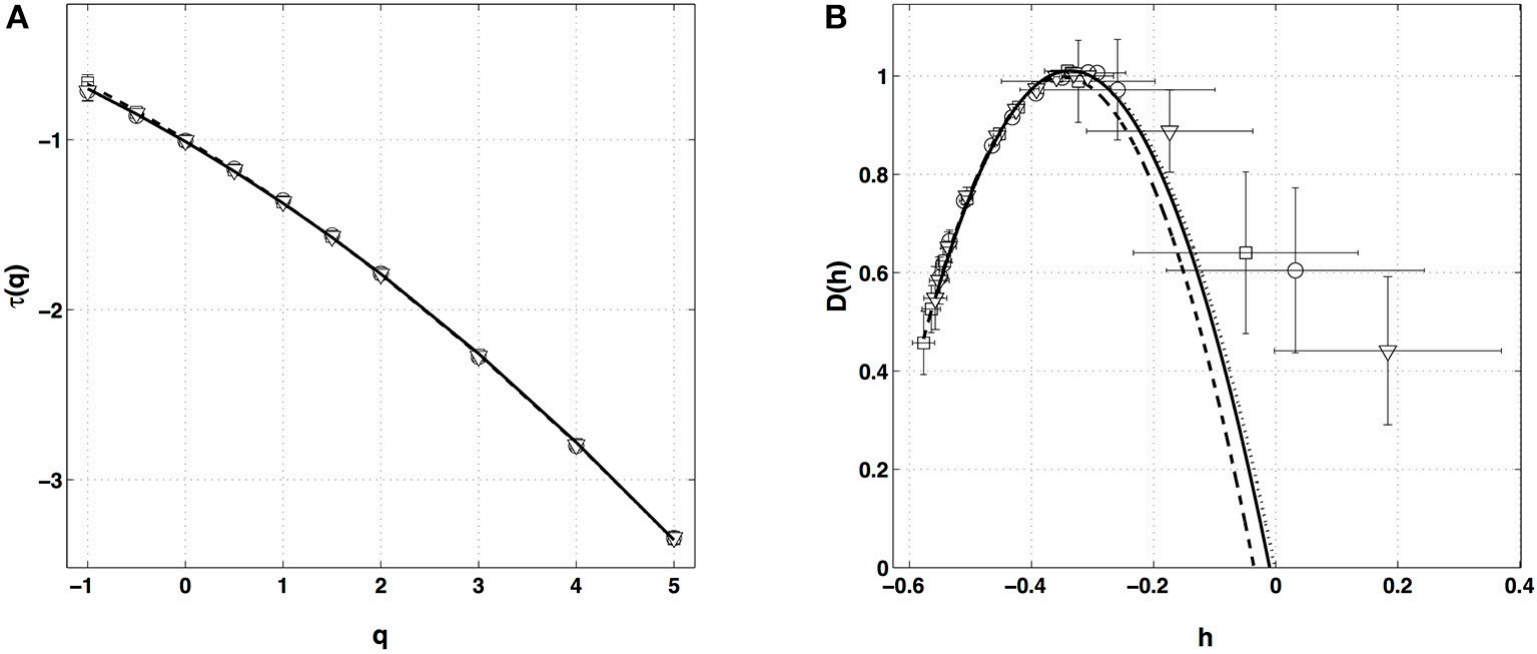
Multifractal spectra of local impulse energy time-series (Pt2) obtained with the WTMM method of moments. **(A)** τ(*q*) vs. *q* estimated by linear regression fit of log_2_ *Z*(*q, a*) vs. log_2_ *a* (Figure 3A). **(B)** *D*(*h*) vs. *h* obtained from linear regression fits of *h*(*q, a*) (Figure 3B) and *D*(*q, a*) (Figure 3C) vs. log_2_ *a*. The symbols correspond to the analyzing wavelets *g*^(1)^ (▿), *g*^(2)^ (□) and *g*^(3)^ (◦) (Figure S1). The curves correspond to quadratic spectra (Equations 10 and 12) with parameters [*c*_0_, *c*_1_, *c*_2_] = [1.01, −0.33, 0.055] (⋯ , *g*^(1)^), [1.00, −0.35, 0.049] (−−−, *g*^(2)^), [1.01, −0.34, 0.053] (—, *g*^(3)^) (see Table 1).

When applying the WTMM method to the impulse energy time-series recorded at Pt2 (Figure 2A), we revealed that the partition functions *Z*(*q, a*) (Equation 3) obtained from the WT computed with the analyzing wavelet *g*^(3)^ (Figure 2B) and its skeleton (Figure 2C), display nice scaling properties for *q* = −1 to 5 over a range of time-scales larger than the mean interbeat ∽0.5 s (Figure 1D). We strictly limited this range to (0.6, 10 s) for linear regression fit estimates in a logarithmic representation (Figure 3A). The τ(*q*) so-obtained is well approximated by a quadratic spectrum with parameters [*c*_0_, *c*_1_, *c*_2_] = [1.01, −0.34, 0.053] and *c*_*n*_ = 0 for *n* > 2 (Equation 10) (Figure 4A). This signature of multifractality with a support of singularities of fractal dimension *D*_*f*_ ≈ 1, and an intermittency coefficient *c*_2_ = 0.053 ± 0.010 (Table 1) is confirmed when respectively plotting *h*(*q, a*)/ln2 (Equation 5) and *D*(*q, a*)/ln2 (Equation 6) vs. log_2_ *a* Figures 3B,C. From the estimate of the slopes *h*(*q*) and *D*(*q*), we get the single humped *D*(*h*) spectrum shown in Figure 4B which is well approximated by the quadratic spectrum defined in Equation (12) with the above parameter values obtained from a polynomial fitting of the τ(*q*) data. Interestingly, when comparing the results obtained with the analyzing wavelet *g*^(3)^ with those obtained with *g*^(1)^ and *g*^(2)^ in Figures 3, 4, we notice that except some slight differences observed when using the first-order analyzing wavelet *g*^(1)^, the multifractal spectra obtained with the second-order wavelet *g*^(2)^ and the third-order wavelet *g*^(3)^ almost superimpose (Figure 4, Table 1) with singularities of Hölder exponent *h* ≤ 0, characteristic of a multifractal “noise” signal.

**Table 1:** Results of the WTMM multifractal analysis of the local impulse energy time-series recorded along the CS vein at electrodes Pt1, Pt2, Pt3, and Pt5.

	***g*^(1)^**	***g*^(2)^**	***g*^(3)^**
**Point1**			
*c*_0_	0.961 ± 0.001	0.995 ± 0.001	1.009 ± 0.002
*c*_1_	−0.351 ± 0.003	−0.298 ± 0.005	−0.281 ± 0.007
$c_{1}^{*}$	−0.353 ± 0.017	−0.297 ± 0.011	−0.274 ± 0.011
*c*_2_	0.048 ± 0.006	0.063 ± 0.011	0.064 ± 0.014
$c_{2}^{*}$	0.050 ± 0.032	0.082 ± 0.018	0.096 ± 0.020
**Point2**			
*c*_0_	1.011 ± 0.002	0.998 ± 0.001	1.011 ± 0.001
*c*_1_	−0.333 ± 0.007	−0.348 ± 0.003	−0.337 ± 0.005
$c_{1}^{*}$	−0.307 ± 0.012	−0.335 ± 0.011	−0.331 ± 0.011
*c*_2_	0.055 ± 0.013	0.049 ± 0.007	0.053 ± 0.010
$c_{2}^{*}$	0.076 ± 0.030	0.031 ± 0.027	0.047 ± 0.028
**Point3**			
*c*_0_	1.023 ± 0.003	1.005 ± 0.002	1.021 ± 0.003
*c*_1_	−0.472 ± 0.014	−0.496 ± 0.008	−0.481 ± 0.011
$c_{1}^{*}$	−0.445 ± 0.011	−0.480 ± 0.008	−0.464 ± 0.007
*c*_2_	0.082 ± 0.028	0.091 ± 0.015	0.098 ± 0.022
$c_{2}^{*}$	0.164 ± 0.031	0.103 ± 0.023	0.164 ± 0.020
**Point5**			
*c*_0_	1.044 ± 0.003	1.017 ± 0.003	1.029 ± 0.002
*c*_1_	−0.320 ± 0.014	−0.365 ± 0.011	−0.383 ± 0.009
$c_{1}^{*}$	−0.256 ± 0.015	−0.335 ± 0.018	−0.365 ± 0.025
*c*_2_	0.176 ± 0.028	0.167 ± 0.022	0.152 ± 0.018
$c_{2}^{*}$	0.175 ± 0.043	0.106 ± 0.029	0.114 ± 0.031

c_0_, c_1_, c_2_ are the coefficients of the polynomial expansion of τ(q) (Equation 10) obtained with the WTMM method of moments when using the analyzing wavelets g^(1)^, g^(2)^, and g^(3)^ respectively (Figure S1). $c_{1}^{*}$ and $c_{2}^{*}$ are the corresponding coefficients obtained with the magnitude cumulant method.

**Figure 5:**
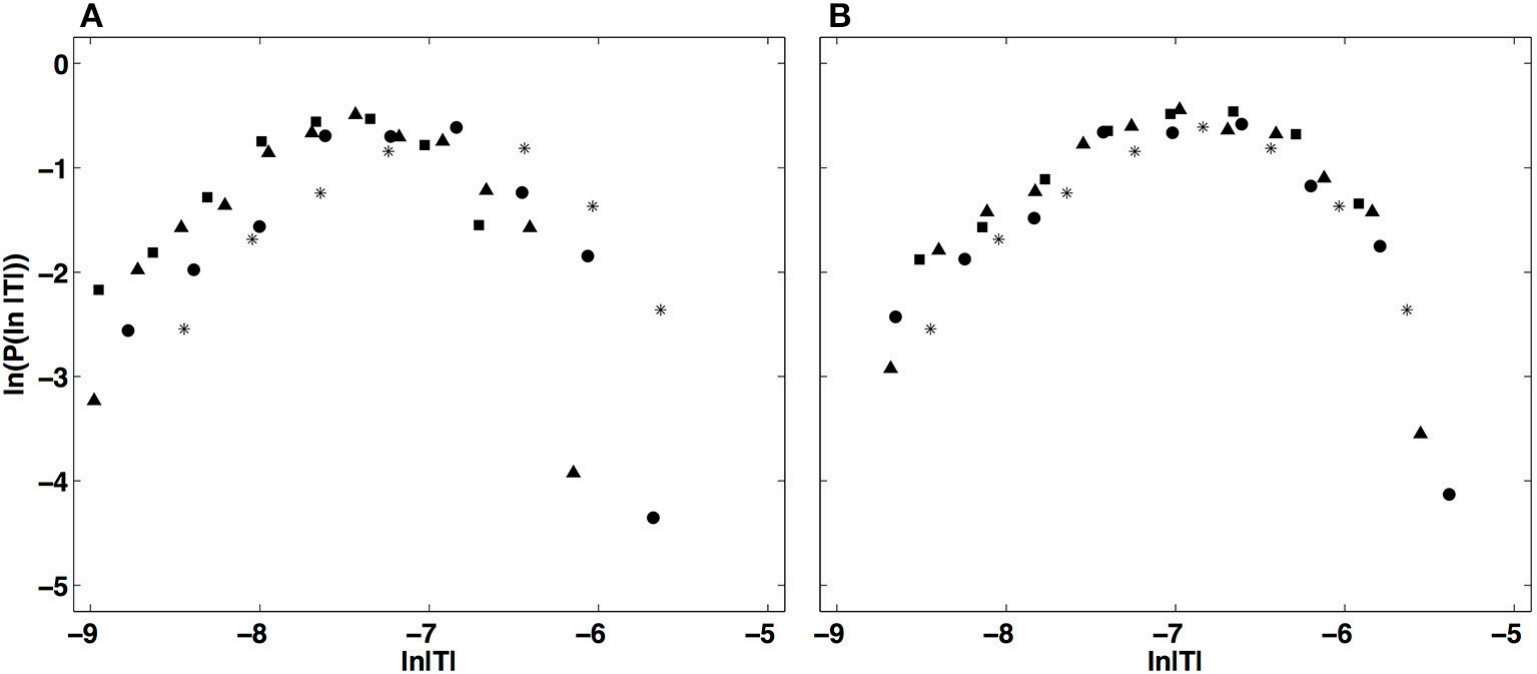
Demonstration of the WTMM pdf rescaling via the propagative equation across scales (Equation 13). **(A)** Original pdfs of the logs of WTMM coefficients (magnitude coefficients) of the local impulse energy time series recorded at the electrode Pt2, computed with the analyzing wavelet *g*^(3)^, at scales *a* (= Δ*t*/Δ*t*_0_, where $\Delta t_{0}=10^{-4}$ s) = 2^9^(^*^), 2^10^(•), 2^11^(▴) and 2^12^(■). **(B)** Rescaled pdfs using the multifractal quadratic estimate of the τ(*q*) spectrum (Equation 10) with parameters [*c*_0_, *c*_1_, *c*_2_] = [1.01, −0.34, 0.053] (see Table 1).

This multifractal diagnosis is confirmed in Figure 5 where the WTMM pdfs obtained at different scales with *g*^(3)^ (Figure 5A) are shown to collapse on each other when using the propagative equation of the statistics across scales (Equation 13) with the quadratic τ(*q*) spectrum estimated just above (Figure 5B) (Castaing et al., 1990, 1993; Arneodo et al., 1997a, 1998c, 1999; Venugopal et al., 2006).

#### 4.1.2. Multifractal analysis of the impulse energy data with the method of magnitude cumulants

**Figure 6:**
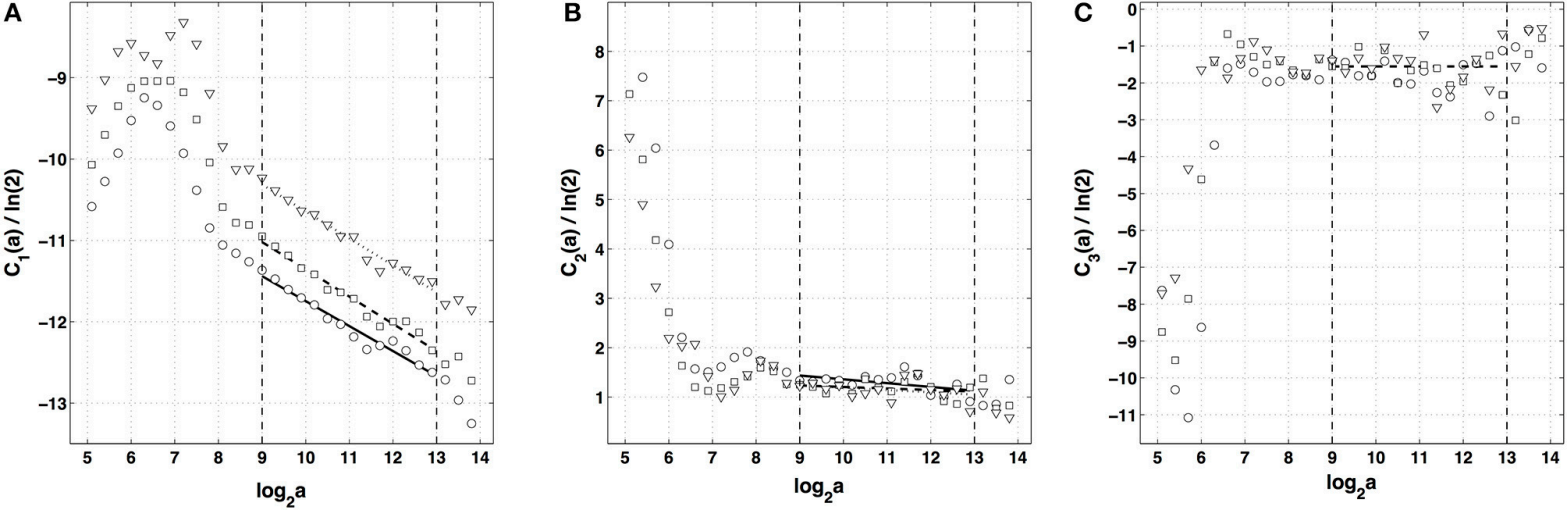
Magnitude cumulant analysis of local impulse energy time-series (Pt2). **(A)** *C*_1_(*a*)/ln 2 vs. log_2_ *a*. **(B)** *C*_2_(*a*)/ln 2 vs. log_2_ *a*. **(C)** *C*_3_(*a*)/ln 2 vs. log_2_ *a*. The computation of the *C*_*n*_(*a*) (Equation 9) was performed with the analyzing wavelets *g*^(1)^ (▿), *g*^(2)^ (□) and *g*^(3)^ (◦) (Figure S1). The vertical dashed lines delimit the range of scales (2^9^ ≤ *a* ≤ 2^13^) used for the linear regression estimate of coefficients $c_{1}^{*}$, $c_{2}^{*}$ and $c_{3}^{*}$ of τ(*q*) (Equation 10) reported in Table 1.

After the WTMM partition function approach, we turn our attention to the alternate magnitude cumulant analysis methodology. The first-, second- and third-order cumulants were computed using Equation (9) and are plotted vs. the logarithm of the scale in Figure 6. As expected *C*_1_(*a*), *C*_2_(*a*) and *C*_3_(*a*) display consistent scaling behavior over the same range of scales 2^9^ ≤ *a* ≤ 2^13^ (*a* = Δ*t*/Δ*t*_0_, where the oversampling time is $\Delta t_{0}=10^{-4}$ s) and this for the three analyzing wavelets *g*^(1)^, *g*^(2)^ and *g*^(3)^. The results obtained for *C*_3_(*a*) (Figure 6C) confirm that with the limited statistical sample at our disposal (422 s long time series), there is no way to conclude about the possible departure from a log-normal quadratic τ(*q*) spectrum (*c*_3_ ≡ 0). Nicely, the quadratic τ(*q*) spectrum obtained with *g*^(3)^ with parameters $c_{1}^{*}=-0.33\pm 0.01$ and $c_{2}^{*}=0.047\pm 0.028$ is found in good agreement with the one previously estimated with the method of moments, confirming the multifractal diagnosis of the local impulse energy at low frequencies. Let us point out that, as reported in Table 1, the τ(*q*) spectrum obtained with *g*^(1)^ is again slightly different from the ones obtained with *g*^(2)^ and *g*^(3)^ which turn out to be indistinguishable. This is the numerical demonstration that a robust estimate of the multifractal spectra is achieved when using the third-order analyzing wavelet *g*^(3)^.

#### 4.1.3. Summary of one-point multifractal analysis of the impulse energy data

**Figure 7:**
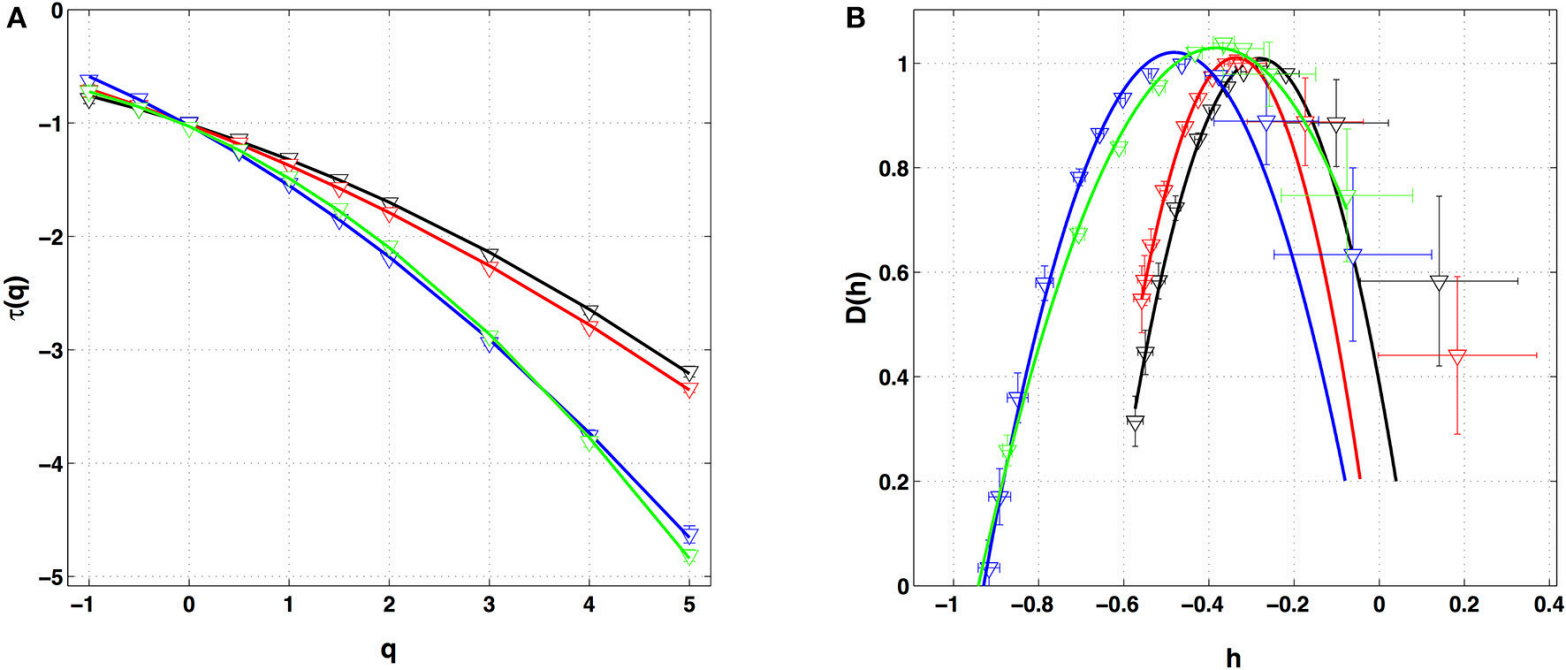
Multifractal spectra of local impulse energy time-series recorded along the CS vein. **(A)** τ(*q*) vs. *q* estimated by linear regression fit of log_2_ *Z*(*q, a*) vs. log_2_ *a*. **(B)** *D*(*h*) vs. *h* obtained from linear regression fits of *h*(*q, a*) and *D*(*q, a*) vs. ln_2_ *a*. The analyzing wavelets is *g*^(3)^. The colored symbols correspond to the electrodes Pt1 (black), Pt2 (red), Pt3 (blue) and Pt5 (green). The curves correspond to quadratic spectra (Equations 10 and 12) with parameters [*c*_0_, *c*_1_, *c*_2_] = [1.01, −0.28, 0.064] (black, Pt1), [1.01, −0.34, 0.053] (red, Pt2), [1.02, −0.48, 0.098] (blue, Pt3), and [1.03, −0.38, 0.152] (green, Pt5) (see Table 1).

**Figure 8:**
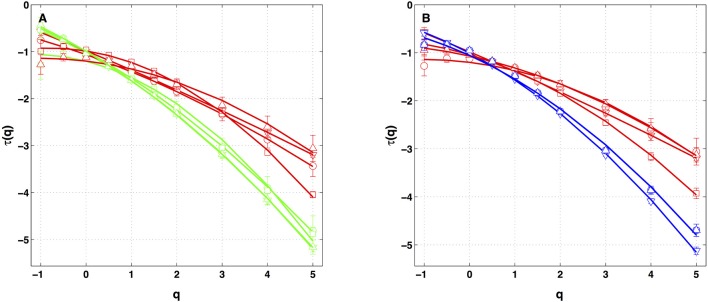
τ(*q*) spectra of local impulse energy time-series recorded along the CS vein at the electrodes Pt2 (red), Pt3 (blue) and Pt5 (green). The curves represent quadratic polynomial fit of the data (Equation 10). **(A)** The symbols correspond to the reference Patient 1 (chronic AF, ▿) and to Patients 2 (chronic AF, ◦), 3 (paroxysmal AF, □) and 4 (persistent AF, △). **(B)** The symbols correspond to the reference Patient 1 (▿) and to three different time-series for Patient 4 (◦, □, △) recorded at different periods of time preceding ablation procedure.

Similar analysis was performed on the four other local impulse energy time-series recorded at electrodes Pt1 (Figures S4, S8, S11), Pt3 (Figures S5, S9, S12), Pt4 (Figures S6, S13), and Pt5 (Figures S7, S10, S14). As already noticeable on the power spectrum in Figure 1D, the electric potential Δϕ(*t*) recorded at electrode Pt4 presents some subharmonic oscillatory component at frequency ∽2.5 Hz that dramatically spoils the scaling behavior previously obtained at Pt2 over the range of time-scales (0.6, 10 s) (Figures S6, S13). We can speculate on the signature of the nearby mitral valve influence. This explains that Pt4 will be singled out in the rest of our study. Figure 7 displays the τ(*q*) (Figure 7A) and *D*(*h*) (Figure 7B) spectra of the local impulse energy *E*(*t*) obtained with the WTMM method of moments when using the analyzing wavelet *g*^(3)^ (the corresponding spectra obtained with analyzing wavelets *g*^(1)^ and *g*^(2)^ are shown in Figures S15, S16 respectively). The spectra obtained for the time-series recorded at electrode Pt1 situated, as Pt2, in the ligament of Marshall anatomic area innervated by the ANS (Figure 1A), are very similar to the ones observed at Pt2. Both τ(*q*) and *D(h)* spectra are well approximated by quadratic spectra (Equations 9 and 10, respectively) with parameters [*c*_0_, *c*_1_, *c*_2_] = [1.01, −0.28, 0.064] (Figure 7) with a definitely positive finite intermittency coefficient *c*_2_ = 0.064 ± 0.014 (Table 1). Interestingly, time series recorded at electrodes Pt3 and Pt5 in a different anatomical area next to the left atrial posterior wall (Figure 1A) both show rather similar τ(*q*) and *D*(*h*) multifractal spectra but significantly different from the ones obtained at electrodes Pt1 and Pt2 (Figure 7). Again these spectra are found nearly quadratic with parameters [*c*_0_, *c*_1_, *c*_2_] = [1.02, −0.48, 0.098] for Pt3, and [1.03, −0.38, 0.152] for Pt5 (Table 1). Local impulse Local impulse energy time series at Pt3 and Pt5 show higher intermittency with larger *c*_2_ values whereas they display weaker long-range correlations *c*_1_ = < *h* > ∽ − 0.45 (i.e., closer to *c*_1_ = −0.5 a value characteristic of uncorrelated white noise), instead of the value *c*_1_∽ − 0.3 for Pt1 and Pt2 characteristic of positive long-range correlations. As reported in Table 1, this regionalization of the multifractal properties of the local impulse energy is quantitatively confirmed when using the magnitude cumulant method. It is further corroborated when reproducing this multifractal analysis for other patients with paroxysmal, persistent or chronic AF (Figure 8A) and for a patient at different periods of time preceding ablation procedure (Figure 8B) as an indication of stationarity.

### 4.2. Two-point magnitude analysis of local impulse energy data

**Figure 9:**
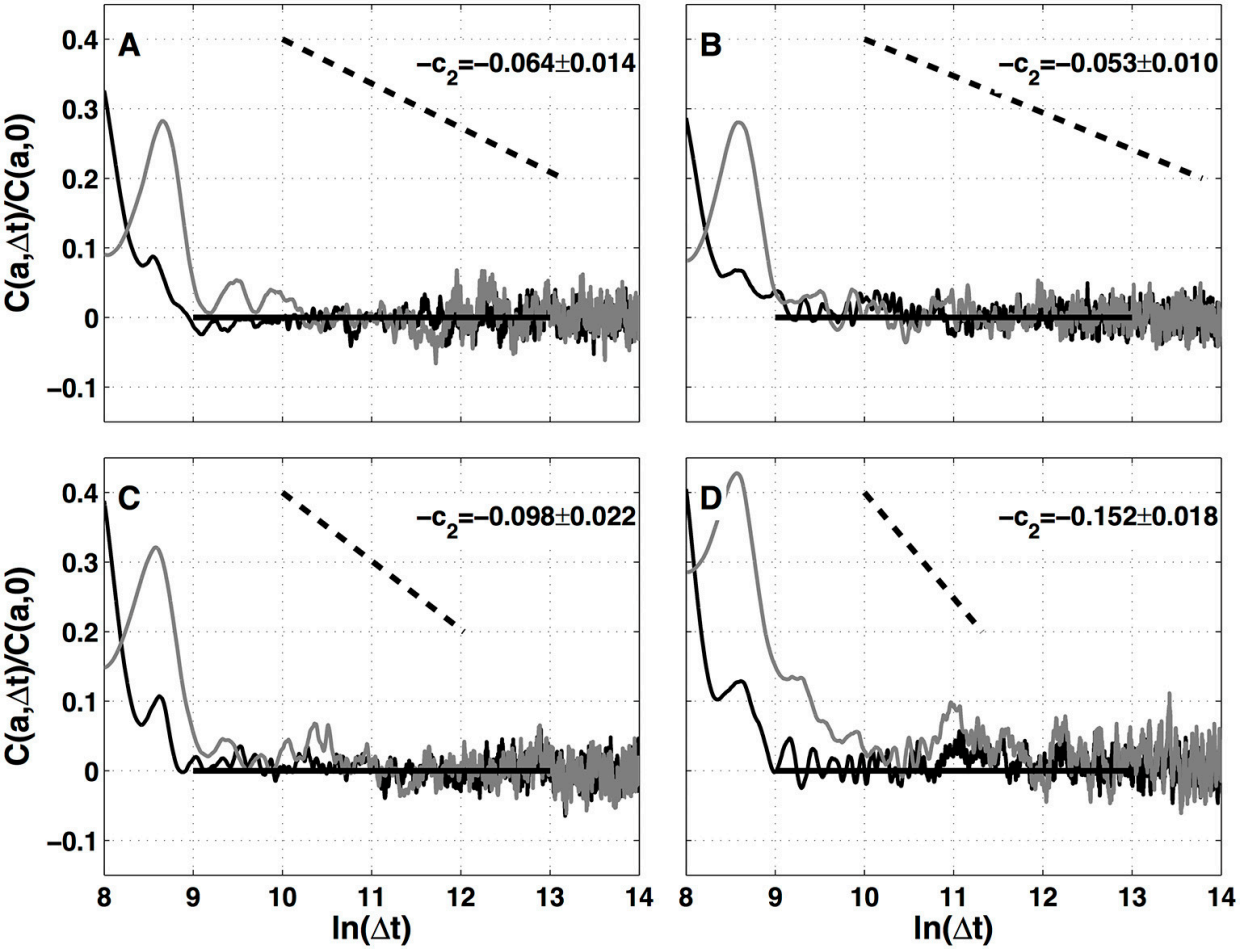
Two-point magnitude analysis of local impulse energy time-series recorded along the CS vein. Two-point correlation function *C*(*a*, Δ*t*)/*C*(*a*, 0) vs. ln (Δ*t*) (Equation 15) for local impulse energy *E*(*t*) computed with the analyzing wavelet *g*^(3)^. The two curves correspond to scales *a* = 2^9^ (black) and 2^10^ (gray) within the scaling range. **(A)** Pt1, **(B)** Pt2, **(C)** Pt3, and **(D)** Pt5.

The results of the two-point magnitude correlation analysis of the local impulse energy time series recorded at the positions Pt1, Pt2, Pt3, and Pt5 along the CS vein are shown in Figure 9. *C*(*a*, Δ*t*) (Equation 15) computed with the analyzing wavelet *g*^(3)^ is represented vs. Δ*t* for two scales *a* = 2^9^ and 2^10^ in the scaling range. Strikingly for all four time series, for Δ*t* ≳ *a*, *C*(*a*, Δ*t*) drops to zero as a clear indication that the magnitudes are uncorrelated. As a reference, we put in each panel of Figure 9, a dashed straight line of slope −*c*_2_ as predicted by Equation (16) for multifractal signals exhibiting a cascading multiplicative structure along a time-scale tree (Arneodo et al., 1998b). The slow decay predicted by the “multiplicative” log-normal model with intermittency coefficient *c*_2_ is definitely not observed. Thus, local impulse energy time-series look much more like what has been called log-normal “mutlifractal white noise” in pioneering works to distinguish “uncorrelated” and “multiplicative” log-normal models (Arneodo et al., 1998a). A similar absence of magnitude correlation is observed when reproducing this two-point magnitude analysis with the analyzing wavelets *g*^(1)^ (Figure S17) and *g*^(2)^ (Figure S18).

## 5. Discussion

To summarize, we showed that the wavelet-based multifractal analysis of long time series of the local impulse energy recorded in the CS of a patient with chronic AF was able to reveal and quantify the intermittent nature of these signals at low frequency (*f* ≲ 2 Hz). To our knowledge, our study is the first to report on the observation and quantification of such multifractal dynamics of the endocavitary electrical activity during chronic AF which is found more complex than previously suspected. On the basis of the analysis of the time-series recorded at 4 catheter electrodes out of 5 positioned in the CS, two main observations can be made: (i) the local impulse energy displays different multifractal properties in the left atrial wall area than in the ligament of Marshall area consistently with different anatomical substrate conditions, and (ii) while recorded along the CS vein, the local impulse energy does not exhibit long-range dependence associated with an underlying multiplicative cascade, or in other words the multifractal distribution of the singularities inferred by the two-point magnitude analysis does not display any correlation across scales just like a log-normal “multifractal white noise” (Arneodo et al., 1998a). The nature of this study was exploratory, with a data set limited to a few patients, and with a few time series rather long for clinical practice (422 s) but not so long regarding the range of time scales [0.6, 10 s] where scaling was observed. This is the reason for the different complementary analyses employed in this paper including the WTMM method of moments, the WTMM method of magnitude cumulants, and the two-points magnitude cumulant method, using analyzing wavelets of different orders, until reliable estimates were obtained. Of course our results deserve to be confirmed over a large set of patients at different stages of AF development and to be explored in different areas of the atria. The goal would be to exhibit precise determinants of the diseased substrate using multifractal scaling analysis. But this preliminary analysis definitely challenges current knowledge in physical, physiological and clinical fundamentals of AF arrhythmia. Specifically, it challenges the mechanistic approach of AF based on circuit reentries.

The absence of an underlying cascading process is not such a surprise since underlying the multifractal properties displayed by the local impulse energy at low frequencies (*f* ≲ 2 Hz), there is no clear 3D “fragmentation” (Mandelbrot, 1982) process inducing some cascading of energy from large to small time scales and also no obvious 2D “aggregation, coalescence or growth” (Vicsek, 1989) process bringing energy from small to large time scales. What are the physical and physiological mechanisms that drive the multifractal nature of local impulse energy and give rise to the observed differences according to area is still an open question. Nonetheless, these results already undermine the commonly accepted concepts revolving around circuit reentries, and a fortiori spiral waves, as being basic mechanisms for the onset and perpetuation of AF. The mechanistic “wavelength” criterion indeed conveys the idea that random spatio-temporal dispersion of refractoriness, or more generally of functional properties, leads to random mixing of circuit reentries. The “wavelength” scale adjusts naturally to the typical scale λ of dispersion when it exists *c* × *RP* ≲ λ, as would be the case for Gaussian statistics of dispersion. In that case, the statistics of the local impulse energy remains Gaussian throughout scales. On the contrary, to fit our new observations we have seen that the statistics is not Gaussian and evolves across scales through a log-normal propagation law which accounts for the intermittency observed over the range of a few beat cycles (~0.6 s) to several tens (~10 s) (and possibly more), therefore spanning the whole atria. Although the ligament of Marshall area is highly innervated (Tan et al., 2007; Ulphani et al., 2007; Arora, 2012), it is quite unlikely that modulations by the ANS, that affects heart rate, play a significant role in the intermittent dynamics since the documented three peak frequencies at ∽0.4, ∽0.15, and ∽0.04 Hz (Akselrod et al., 1981) do not show up in our analysis. Furthermore, we have found at least two areas with different multifractal regimes. Thus, our findings raise new challenging questions calling for ongoing efforts to develop physiological heart tissue models that account for the low frequency intermittent nature of local impulse energy. Recent studies in animal models suggest the protective role of connexin gene transfer to prevent sustained AF (Bikou et al., 2011; Igarashi et al., 2012). In this spirit, in a companion modeling paper (Attuel et al., submitted), we propose a model of gap junction conduction remodeling in a denervated heart that accounts for the observed intermittent dynamics over large time scales, as resulting from incoherent random back scatterings leading to the desynchronization of the network of cardiac excitable cells.

## Author contributions

Conception and design: GA, HY, AA. Development and methodology: EG-C, FA, AA. Analysis and interpretation of data: GA, EG-C, FA, HY, AA. Acquisition of data (provided animals, acquired and managed patients, provided facilities, etc.): GA, HY. Writing, review, and/or revision of the manuscript: GA, EG-C, FA, HY, AA. Administrative, technical or material support (i.e., requiring and organizing data, constructing databases): GA, EG-C.

### Conflict of interest statement

The authors declare that the research was conducted in the absence of any commercial or financial relationships that could be construed as a potential conflict of interest.
