# Supplementary material for: Multifractal Desynchronization of the Cardiac Excitable Cell Network During Atrial Fibrillation. I. Multifractal Analysis of Clinical Data
Source: Front Physiol. 2018 Mar 26;8:1139. doi: 10.3389/fphys.2017.01139 (PMC5880174; doi:10.3389/fphys.2017.01139)
Supplement: Supplementary file 1 [file Presentation1.PDF]

Supplementary Material: Multifractal desynchronization of the cardiac excitable cell network during atrial fibrillation. I. Multifractal analysis of clinical data

1 SUPPLEMENTARY TABLES AND FIGURES

1.1 Figures

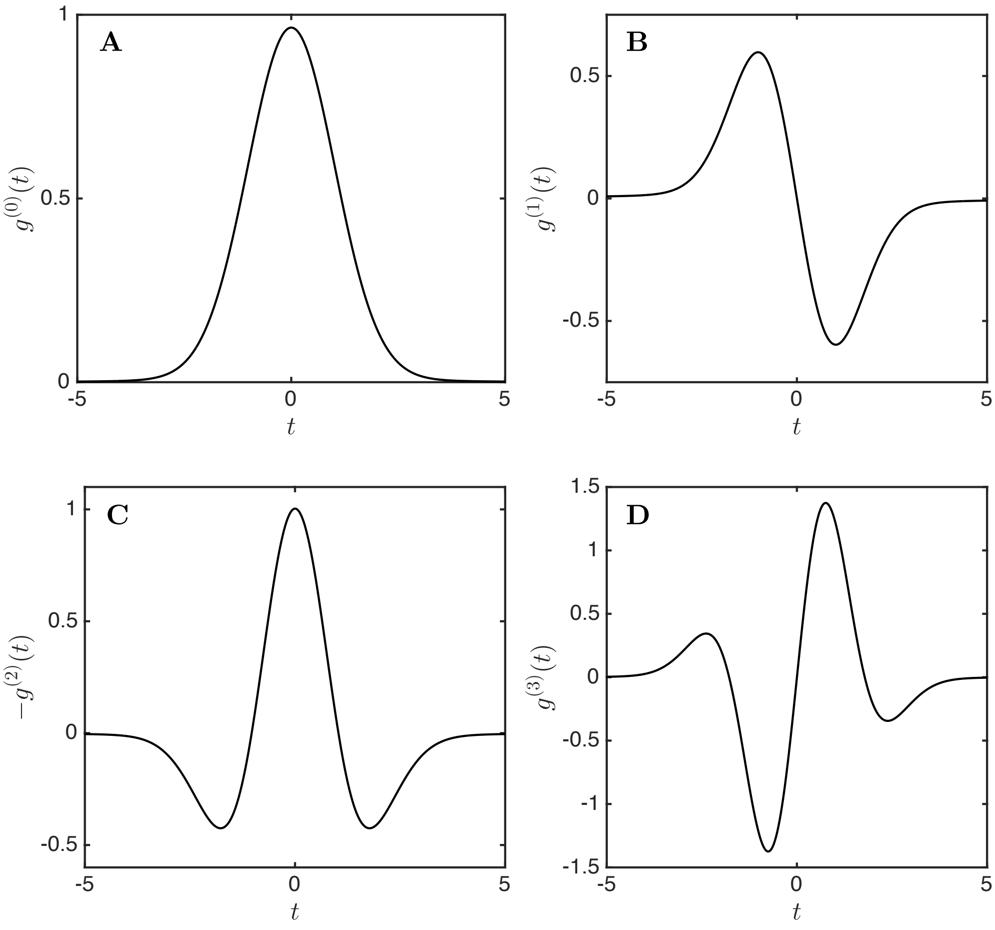

Figure S1: Set of analyzing wavelets defined by the successive derivatives of the Gausssian function $g^{(N)}(t) = \frac{d^N}{dt^N}\left(\exp^{-t^2/2}\right)$. $n_\psi = N$ correspond to the number of vanishing moments. (A) $g^{(0)}(t)$. (B) $g^{(1)}(t)$. (C) $g^{(2)}(t)$. (D) $g^{(3)}(t)$.

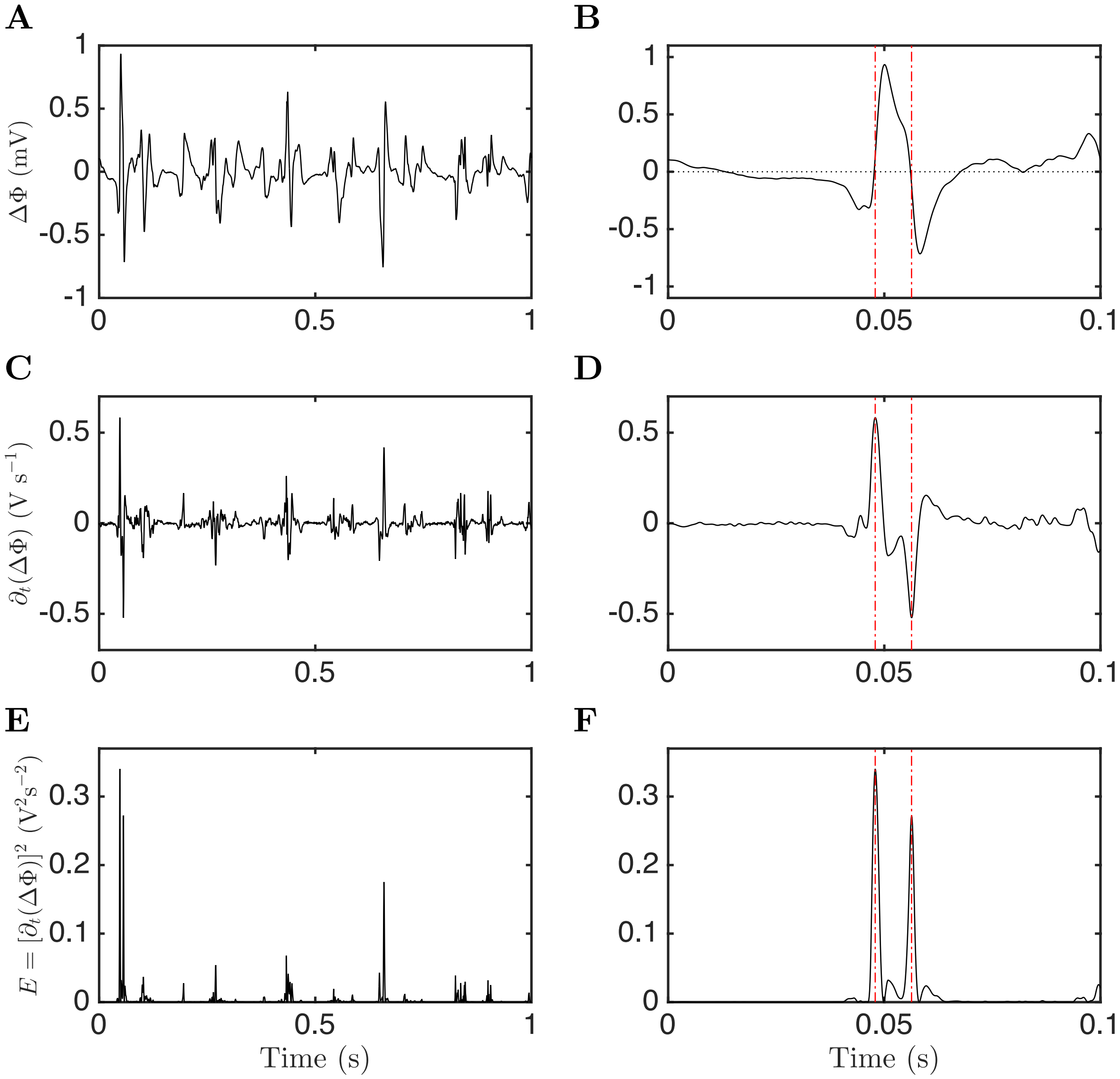

Figure S2: From the recorded electrical potential $\Delta\phi(t)$ to the local impulse energy $E(t)$ (Eq.(17)). (A) A 1 s portion of $\Delta\phi(t)$ (in mv unit) recorded at the electrode Pt2. (B) Zoom on (A) showing the characteristic time scale $\sim 10^{-2}$ s of the local electrical impulse. (C) First derivative of $\Delta\phi(t)$ computed with an order 4 finite difference scheme with a sampling time $\Delta t = 10^{-3}$ s. (D) Zoom on (C). (E) Corresponding energy $E(t) = [\partial_t \Delta\phi(t)]^2$ (V^2 s^{-2}) time series. (F) Zoom on (E).

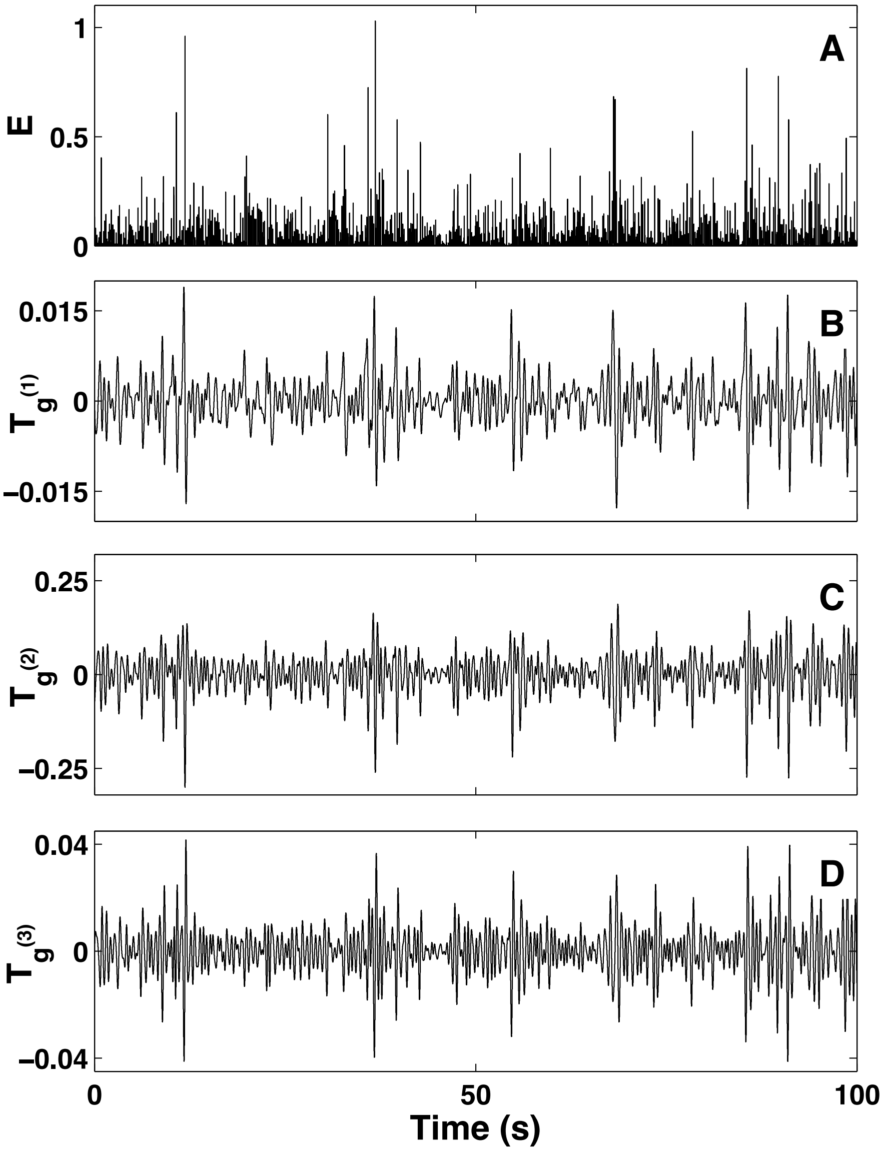

Figure S3: Wavelet transform of local impulse energy $E(t)$ at scale $a^* = 2^9$ in 10^{-4} s unit. (A) A 100 s portion of $E(t)$ recorded at electrode Pt2. (B) $T_{g^{(1)}}(t, a^*)$ *vs* t. (C) $T_{g^{(2)}}(t, a^*)$ *vs* t. (d) $T_{g^{(3)}}(t, a^*)$ *vs* t. The analyzing wavelets are defined in Figure S1.

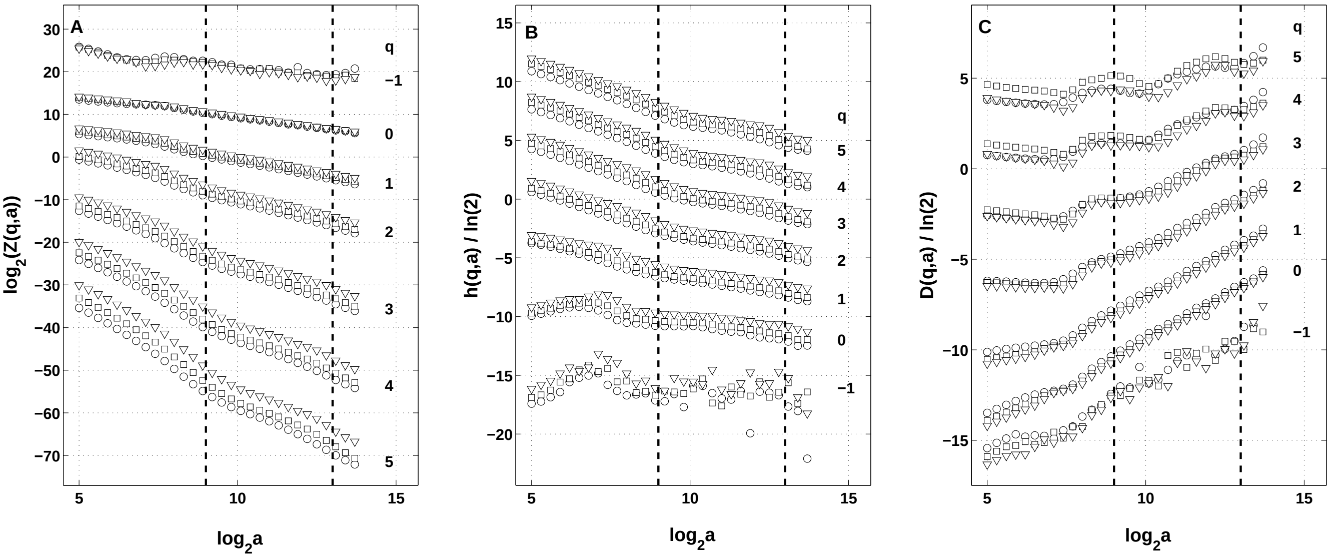

Figure S4: Multifractal analysis of local impulse energy time-series recorded with the electrode Pt1 with the WTMM method. (A) $\log_2 Z(q,a)$ *vs* $\log_2 a$ (Eq. (3)). (B) $h(q,a)/\ln 2$ *vs* $\log_2 a$ (Eq. (5)). (C) $D(q,a)/\ln 2$ *vs* $\log_2 a$ (Eq. (6)). The computation were performed for different values of $q = -1$ to 5 with the analyzing wavelet $g^{(1)}$ ($\bigtriangledown$), $g^{(2)}$ ($\square$) and $g^{(3)}$ ($\circ$) (Figure S1). The vertical dashed lines delimit the range of scale ($2^9 \leq a \leq 2^{13}$) expressed in 10^{-4} s unit, used for the linear regression estimate of $\tau(q)$, $h(q)$ and $D(q)$ in Figure S8.

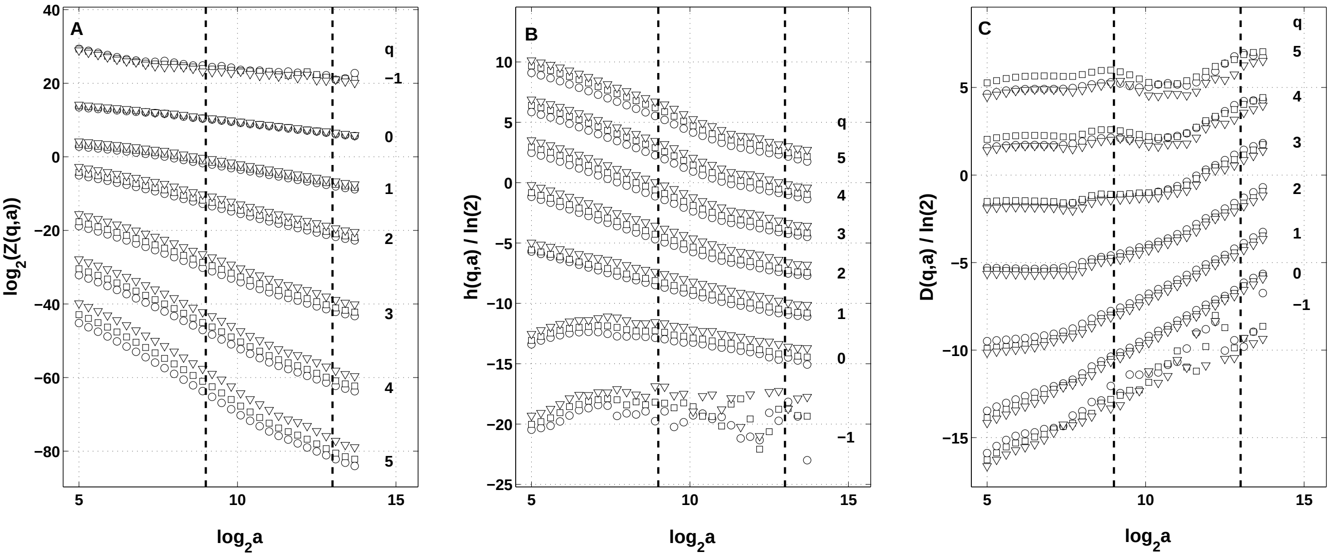

Figure S5: Multifractal analysis of local impulse energy time-series recorded with the electrode Pt3 with the WTMM method. (A) $\log_2 Z(q,a)$ *vs* $\log_2 a$ (Eq. (3)). (B) $h(q,a)/\ln 2$ *vs* $\log_2 a$ (Eq. (5)). (C) $D(q,a)/\ln 2$ *vs* $\log_2 a$ (Eq. (6)). The computation were performed for different values of $q = -1$ to 5 with the analyzing wavelet $g^{(1)}$ ($\triangledown$), $g^{(2)}$ ($\Box$) and $g^{(3)}$ ($\circ$) (Figure S1). The vertical dashed lines delimit the range of scale ($2^9 \leq a \leq 2^{13}$) expressed in 10^{-4} s unit, used for the linear regression estimate of $\tau(q)$, $h(q)$ and $D(q)$ in Figure S9.

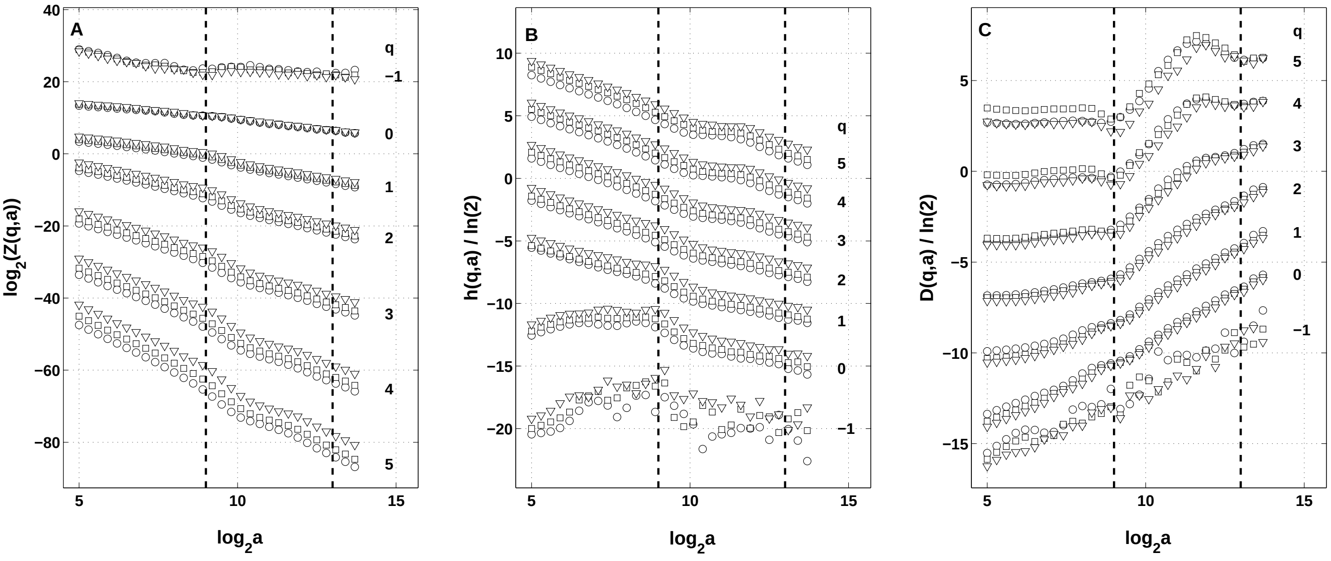

Figure S6: Multifractal analysis of local impulse energy time-series recorded with the electrode Pt4 with the WTMM method. (A) $\log_2 Z(q,a)$ *vs* $\log_2 a$ (Eq. (3)). (B) $h(q,a)/\ln 2$ *vs* $\log_2 a$ (Eq. (5)). (C) $D(q,a)/\ln 2$ *vs* $\log_2 a$ (Eq. (6)). The computation were performed for different values of $q = -1$ to 5 with the analyzing wavelet $g^{(1)}$ ($\triangledown$), $g^{(2)}$ ($\square$) and $g^{(3)}$ ($\circ$) (Figure S1). The vertical dashed lines delimit the range of scale ($2^9 \leq a \leq 2^{13}$) expressed in 10^{-4} s unit, used for the linear regression estimate of $\tau(q)$, $h(q)$ and $D(q)$. As compared to the time series recorded at the electrodes Pt1, Pt2, Pt3 and Pt5, the scaling is spoiled by the presence of a subharmonic interbeat component clearly seen in Figure 1D.

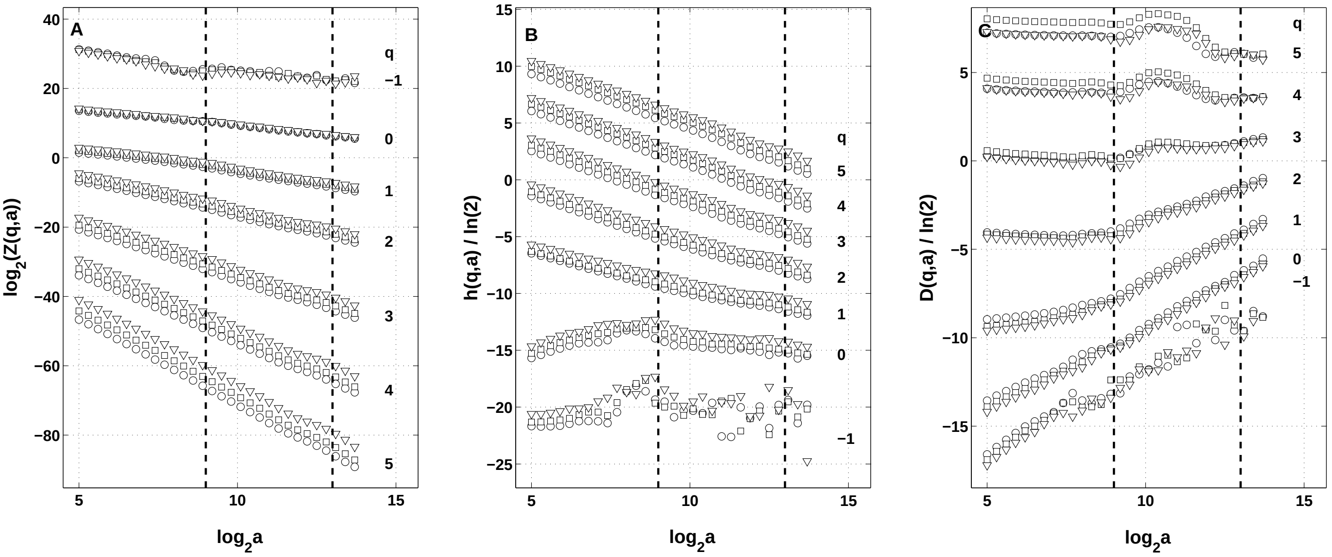

Figure S7: Multifractal analysis of local impulse energy time-series recorded with the electrode Pt5 with the WTMM method. (A) $\log_2 Z(q,a)$ *vs* $\log_2 a$ (Eq. (3)). (B) $h(q,a)/\ln 2$ *vs* $\log_2 a$ (Eq. (5)). (C) $D(q,a)/\ln 2$ *vs* $\log_2 a$ (Eq. (6)). The computation were performed for different values of $q = -1$ to 5 with the analyzing wavelet $g^{(1)}$ ($\triangledown$), $g^{(2)}$ ($\square$) and $g^{(3)}$ ($\circ$) (Figure S1). The vertical dashed lines delimit the range of scale ($2^9 \leq a \leq 2^{13}$) expressed in 10^{-4} s unit, used for the linear regression estimate of $\tau(q)$, $h(q)$ and $D(q)$ in Figure S10.

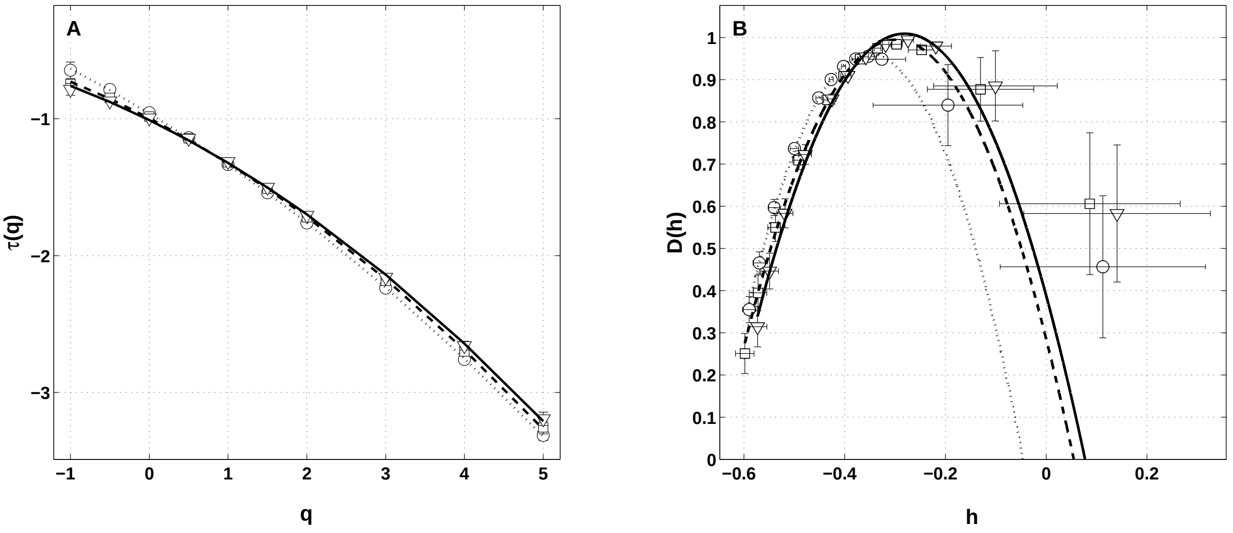

Figure S8: Multifractal spectra of local impulse energy time series recorded at the electrode Pt1. (A) $\tau(q)$ vs q estimated by linear regression fit of $\log_2 Z(q,a)$ *vs* $\log_2 a$ (Figure S4A). (B) $D(h)$ *vs* h obtained from linear regression fits of $h(q,a)$ (Figure S4B) and $D(q,a)$ (Figure S4C) *vs* $\log_2 a$. The symbols correspond to the results obtained with the WTMM of moments when using the analyzing wavelets $g^{(1)}$ ($\triangledown$), $g^{(2)}$ ($\square$) and $g^{(3)}$ ($\circ$) (Figure S1). The curves correspond to quadratic spectra (Eqs (10) and (11)) with parameters $[c_0, c_1, c_2] = [0.96, -0.35, 0.048]$ ($\cdots$, $g^{(1)}$), $[c_0, c_1, c_2] = [1.00, -0.30, 0.063]$ ($- - -$, $g^{(2)}$), $[c_0, c_1, c_2] = [1.01, -0.28, 0.064]$ (——, $g^{(3)}$) (see Table 1).

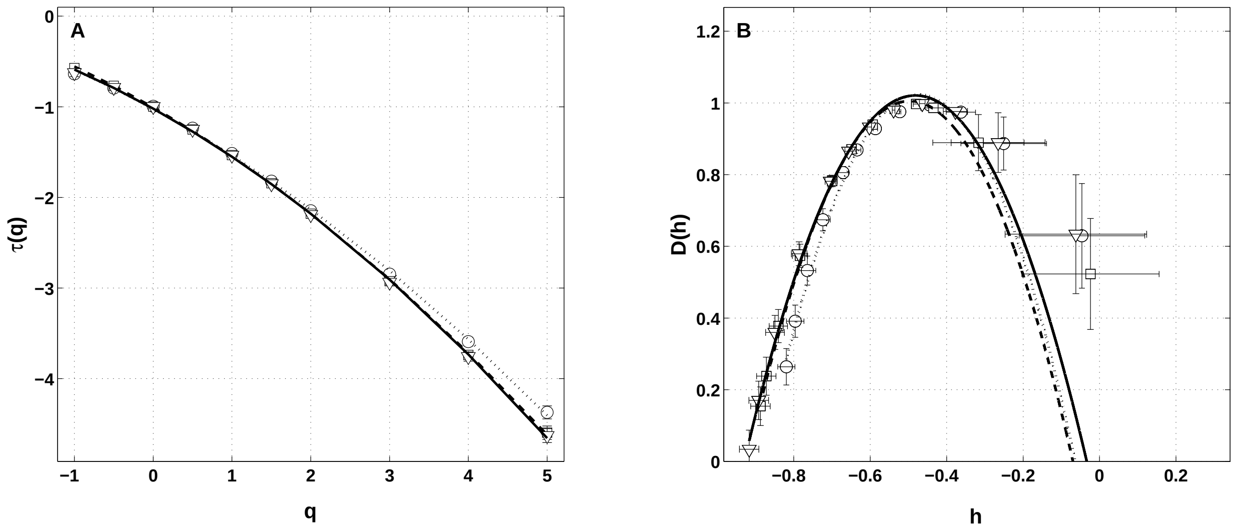

Figure S9: Multifractal spectra of local impulse energy time series recorded at the electrode Pt3. (A) $\tau(q)$ vs q estimated by linear regression fit of $\log_2 Z(q,a)$ *vs* $\log_2 a$ (Figure S5A). (B) $D(h)$ *vs* h obtained from linear regression fits of $h(q,a)$ (Figure S5B) and $D(q,a)$ (Figure S5C) *vs* $\log_2 a$. The symbols correspond to the results obtained with the WTMM of moments when using the analyzing wavelets $g^{(1)}$ ($\triangledown$), $g^{(2)}$ ($\square$) and $g^{(3)}$ ($\circ$) (Figure S1). The curves correspond to quadratic spectra (Eqs (10) and (11)) with parameters $[c_0, c_1, c_2] = [1.02, -0.47, 0.082]$ ($\cdots$, $g^{(1)}$), $[c_0, c_1, c_2] = [1.05, -0.50, 0.091]$ ($- - -$, $g^{(2)}$), $[c_0, c_1, c_2] = [1.02, -0.48, 0.098]$ (——, $g^{(3)}$) (see Table 1).

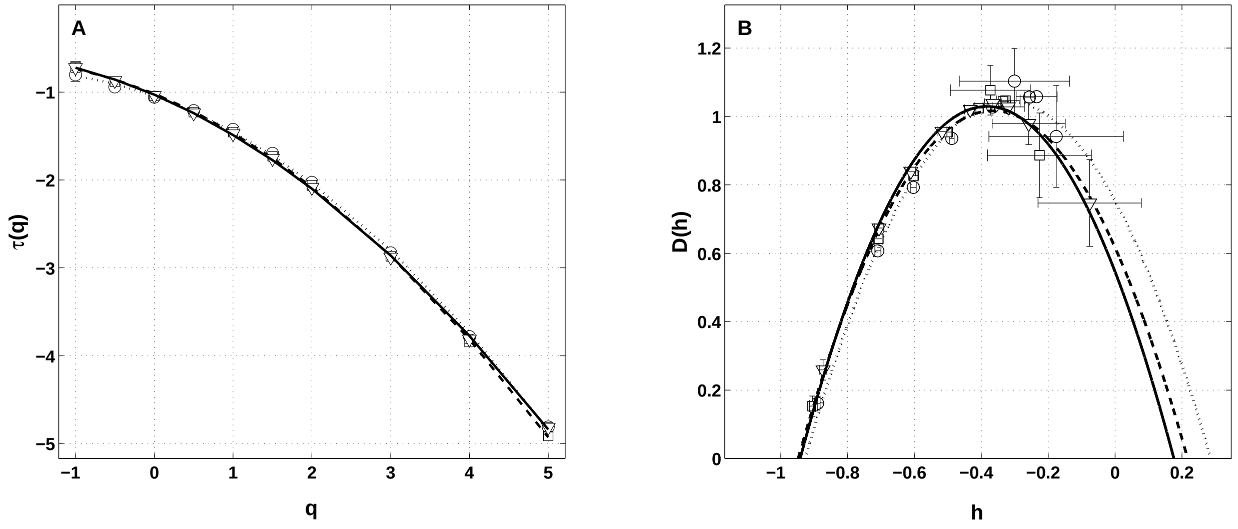

Figure S10: Multifractal spectra of local impulse energy time series recorded at the electrode Pt5. (A) $\tau(q)$ vs q estimated by linear regression fit of $\log_2 Z(q,a)$ *vs* $\log_2 a$ (Figure S7A). (B) $D(h)$ *vs* h obtained from linear regression fits of $h(q,a)$ (Figure S7B) and $D(q,a)$ (Figure S7C) *vs* $\log_2 a$. The symbols correspond to the results obtained with the WTMM of moments when using the analyzing wavelets $g^{(1)}$ ($\triangledown$), $g^{(2)}$ ($\square$) and $g^{(3)}$ ($\circ$) (Figure S1). The curves correspond to quadratic spectra (Eqs (10) and (11)) with parameters $[c_0, c_1, c_2] = [1.04, -0.32, 0.176]$ ($\cdots$, $g^{(1)}$), $[c_0, c_1, c_2] = [1.02, -0.37, 0.167]$ ($- - -$, $g^{(2)}$), $[c_0, c_1, c_2] = [1.03, -0.38, 0.152]$ (——, $g^{(3)}$) (see Table 1).

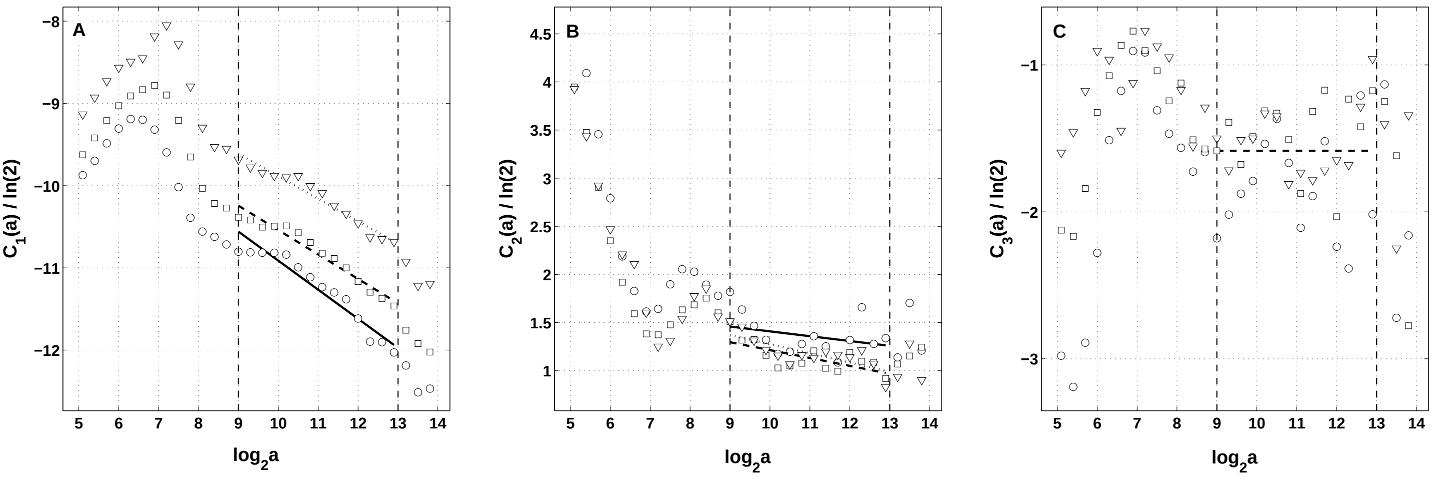

Figure S11: Magnitude cumulant analysis of local impulse energy time series recorded at electrode Pt1. (A) $C_1(a)/\ln 2$ *vs* $\log_2 a$. (B) $C_2(a)/\ln 2$ *vs* $\log_2 a$. (C) $C_3(a)/\ln 2$ *vs* $\log_2 a$. The computation of the $C_n(a)$ (Eq. (9)) was performed with the analyzing wavelets $g^{(1)}$ ($\triangledown$), $g^{(2)}$ ($\square$) and $g^{(3)}$ ($\circ$) (Figure S1). The vertical dashed lines delimit the range of scales ($2^9 \leq a \leq 2^{13}$) expressed in 10^{-4} unit, used for the linear regression estimate of the coefficients c_1^*, c_2^* and c_3^* of $\tau(q)$ (Eq. (10)) reported in Table 1.

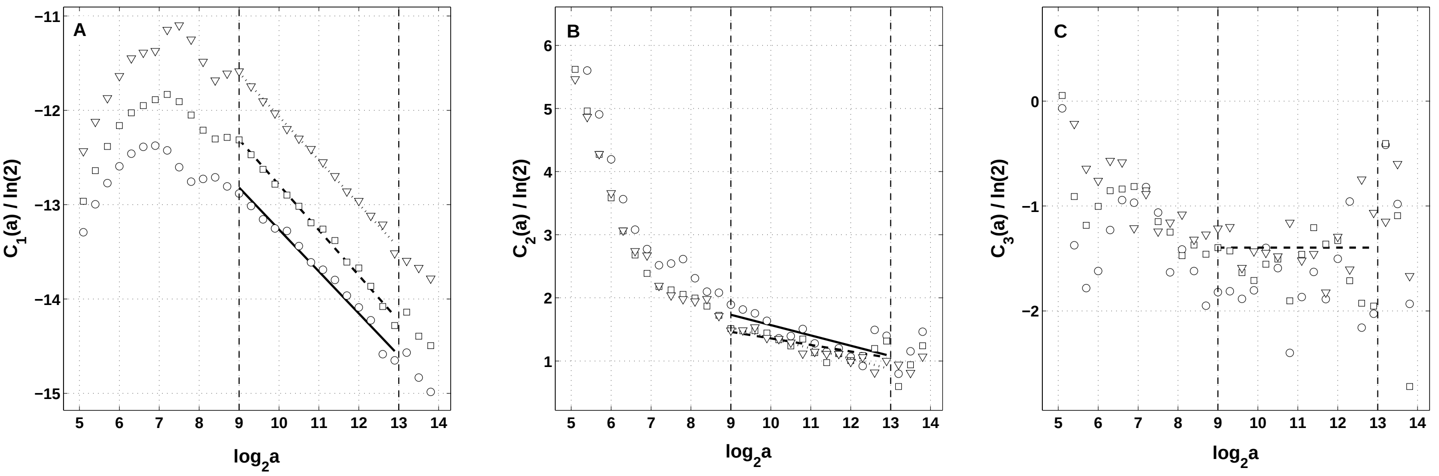

Figure S12: Magnitude cumulant analysis of local impulse energy time series recorded at electrode Pt3. (A) $C_1(a)/\ln 2$ *vs* $\log_2 a$. (B) $C_2(a)/\ln 2$ *vs* $\log_2 a$. (C) $C_3(a)/\ln 2$ *vs* $\log_2 a$. The computation of the $C_n(a)$ (Eq. (9)) was performed with the analyzing wavelets $g^{(1)}$ (∇), $g^{(2)}$ ($\Box$) and $g^{(3)}$ ($\circ$) (Figure S1). The vertical dashed lines delimit the range of scales ($2^9 \leq a \leq 2^{13}$) expressed in 10^{-4} unit, used for the linear regression estimate of the coefficients c_1^*, c_2^* and c_3^* of $\tau(q)$ (Eq. (10)) reported in Table 1.

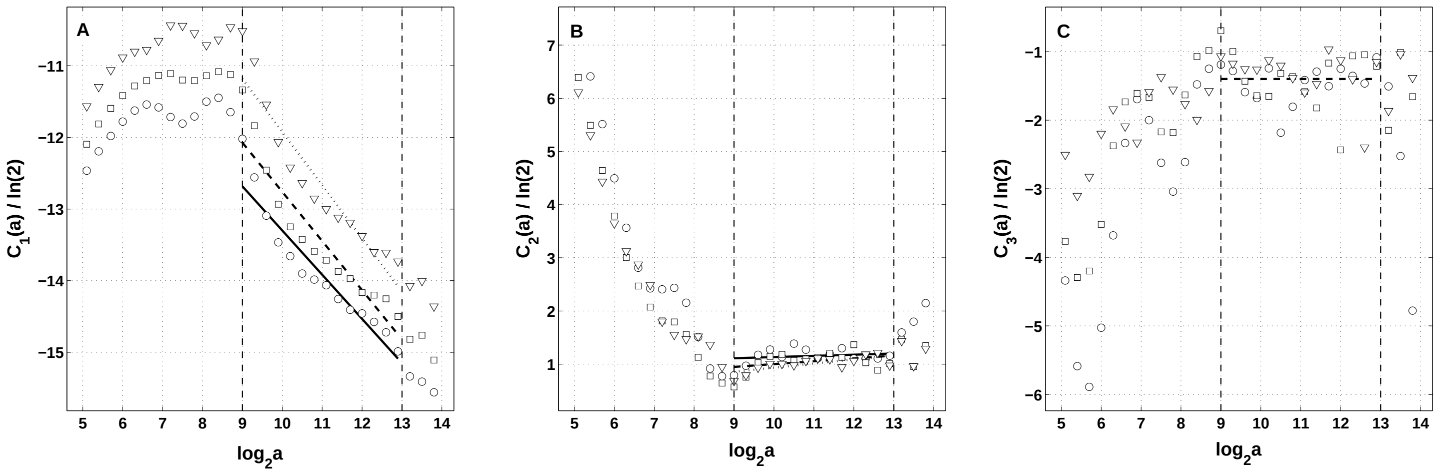

Figure S13: Magnitude cumulant analysis of local impulse energy time series recorded at electrode Pt4. (A) $C_1(a)/\ln 2$ *vs* $\log_2 a$. (B) $C_2(a)/\ln 2$ *vs* $\log_2 a$. (C) $C_3(a)/\ln 2$ *vs* $\log_2 a$. The computation of the $C_n(a)$ (Eq. (9)) was performed with the analyzing wavelets $g^{(1)}$ ($\triangledown$), $g^{(2)}$ ($\square$) and $g^{(3)}$ ($\circ$) (Figure S1). The vertical dashed lines delimit the range of scales ($2^9 \leq a \leq 2^{13}$) expressed in 10^{-4} unit, used for the linear regression estimate of the coefficients c_1^*, c_2^* and c_3^* of $\tau(q)$ (Eq. (10)) reported in Table 1.

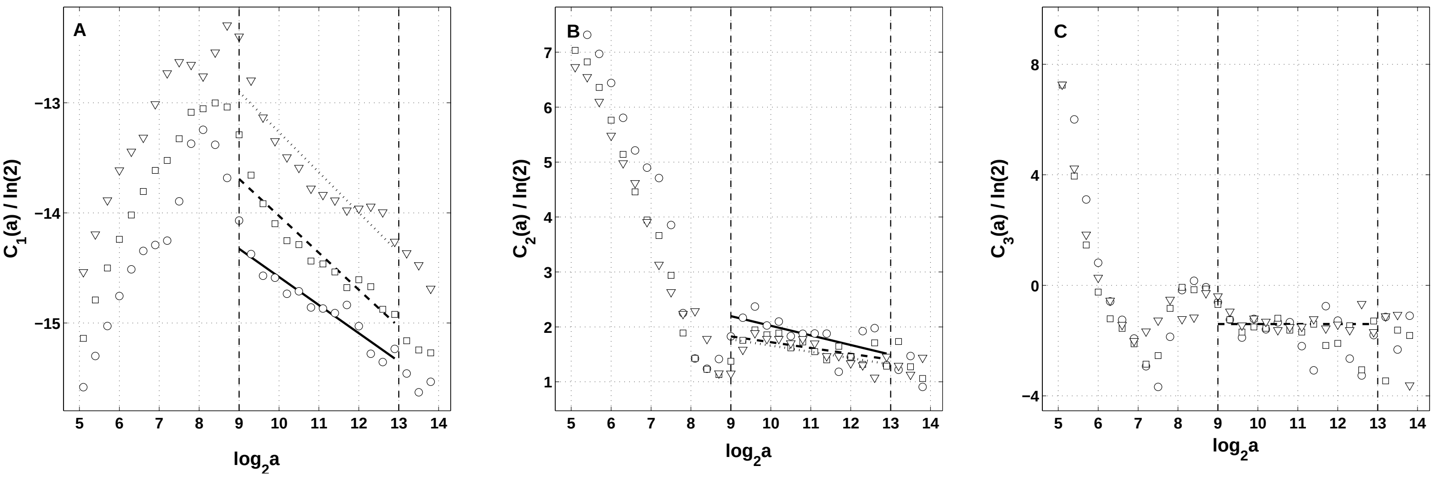

Figure S14: Magnitude cumulant analysis of local impulse energy time series recorded at electrode Pt5. (A) $C_1(a)/\ln 2$ *vs* $\log_2 a$. (B) $C_2(a)/\ln 2$ *vs* $\log_2 a$. (C) $C_3(a)/\ln 2$ *vs* $\log_2 a$. The computation of the $C_n(a)$ (Eq. (9)) was performed with the analyzing wavelets $g^{(1)}$ ($\bigtriangledown$), $g^{(2)}$ ($\Box$) and $g^{(3)}$ ($\circ$) (Figure S1). The vertical dashed lines delimit the range of scales ($2^9 \le a \le 2^{13}$) expressed in 10^{-4} unit, used for the linear regression estimate of the coefficients c_1^*, c_2^* and c_3^* of $\tau(q)$ (Eq. (10)) reported in Table 1.

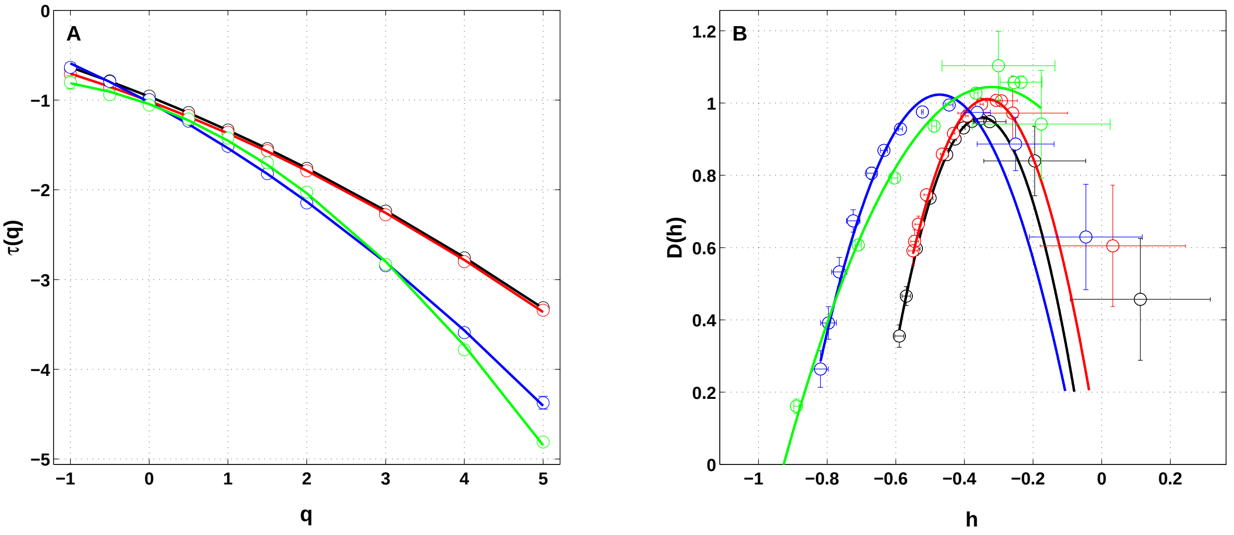

Figure S15: Multifractal spectra of local impulse energy time-series recorded along the CS vein. (A) $\tau(q)$ *vs* q estimated by linear regression fit of $\log_2 Z(q,a)$ *vs* $\log_2 a$. (B) $D(h)$ *vs* h obtained from linear regression fits of $h(q,a)$ and $D(q,a)$ *vs* $\ln_2 a$. The analyzing wavelets is $g^{(1)}$. The colored symbols correspond to electrodes Pt1 (black), Pt2 (red), Pt3 (blue) and Pt5 (green). The curves correspond to quadratic spectra (Eqs (10) and (11)) with parameters $[c_0, c_1, c_2] = [0.96, -0.35, 0.048]$ (black, Pt1), $[1.01, -0.33, 0.055]$ (red, Pt2), $[1.02, -0.47, 0.082]$ (blue, Pt3), and $[1.04, -0.32, 0.176]$ (green, Pt5) (see Table 1).

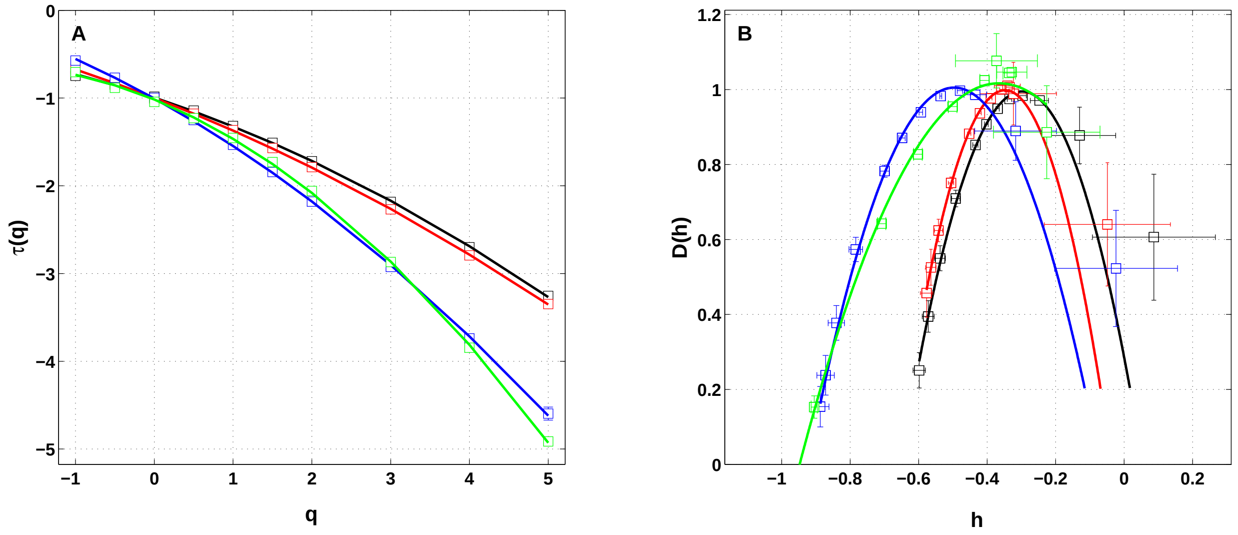

Figure S16: Multifractal spectra of local impulse energy time-series recorded along the CS vein. (A) $\tau(q)$ *vs* q estimated by linear regression fit of $\log_2 Z(q,a)$ *vs* $\log_2 a$. (B) $D(h)$ *vs* h obtained from linear regression fits of $h(q,a)$ and $D(q,a)$ *vs* $\ln_2 a$. The analyzing wavelets is $g^{(2)}$. The colored symbols correspond to electrodes Pt1 (black), Pt2 (red), Pt3 (blue) and Pt5 (green). The curves correspond to quadratic spectra (Eqs (10) and (11)) with parameters $[c_0, c_1, c_2] = [1.00, -0.30, 0.063]$ (black, Pt1), $[1.00, -0.35, 0.049]$ (red, Pt2), $[1.01, -0.50, 0.091]$ (blue, Pt3), and $[1.02, -0.37, 0.167]$ (green, Pt5) (see Table 1).

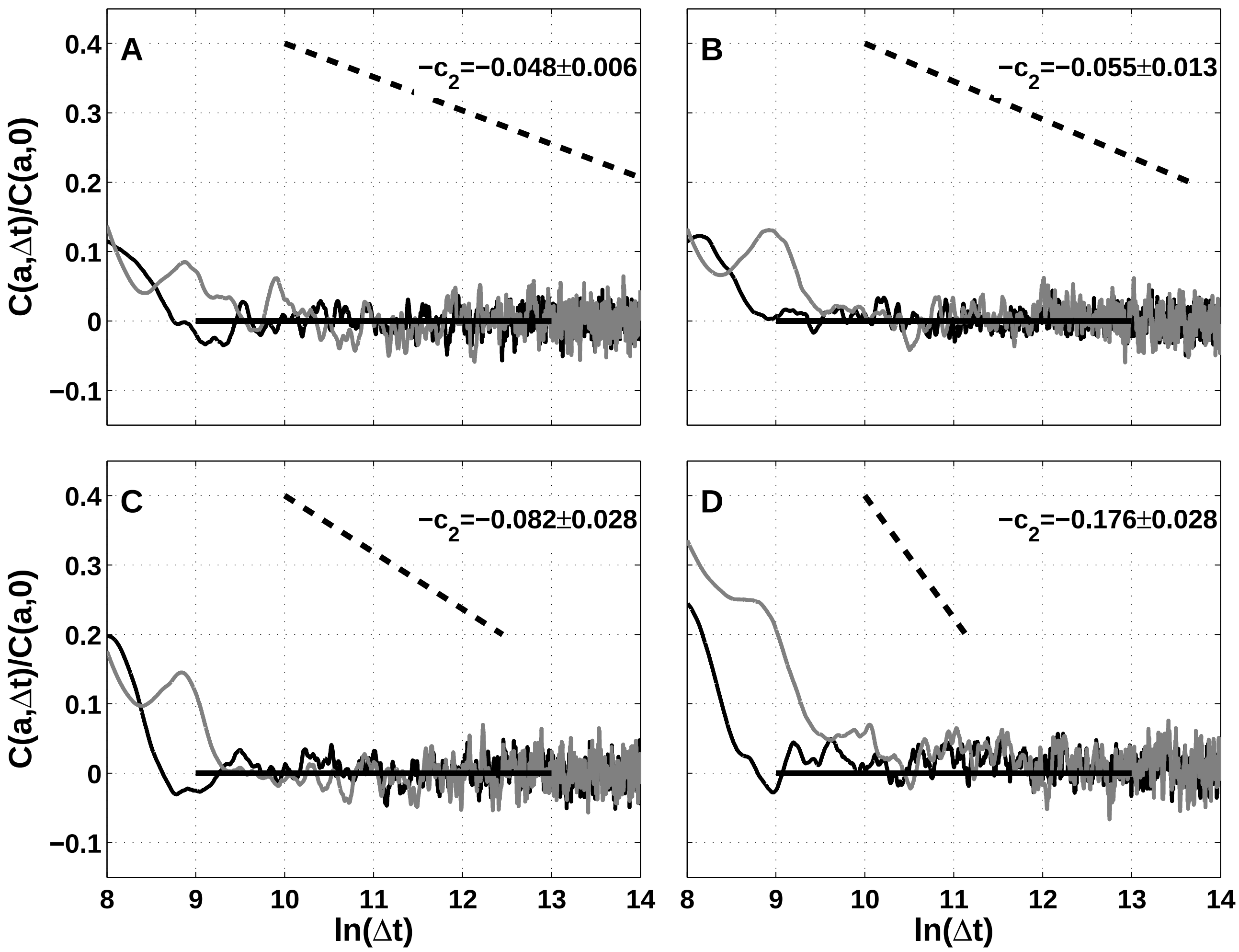

Figure S17: Two-point magnitude analysis of local impulse energy time-series recorded along the CS vein. Two-point correlation function $C(a,\Delta t)/C(a,0)$ *vs* $\ln(\Delta t)$ (Eq. (13)) for local impulse energy $E(t)$ computed with the analyzing wavelet $g^{(1)}$. The two curves correspond to scales $a = 2^9$ (black) and 2^{10} (grey) within the scaling range. (A) Pt1, (B) Pt2, (C) Pt3, and (D) Pt5.

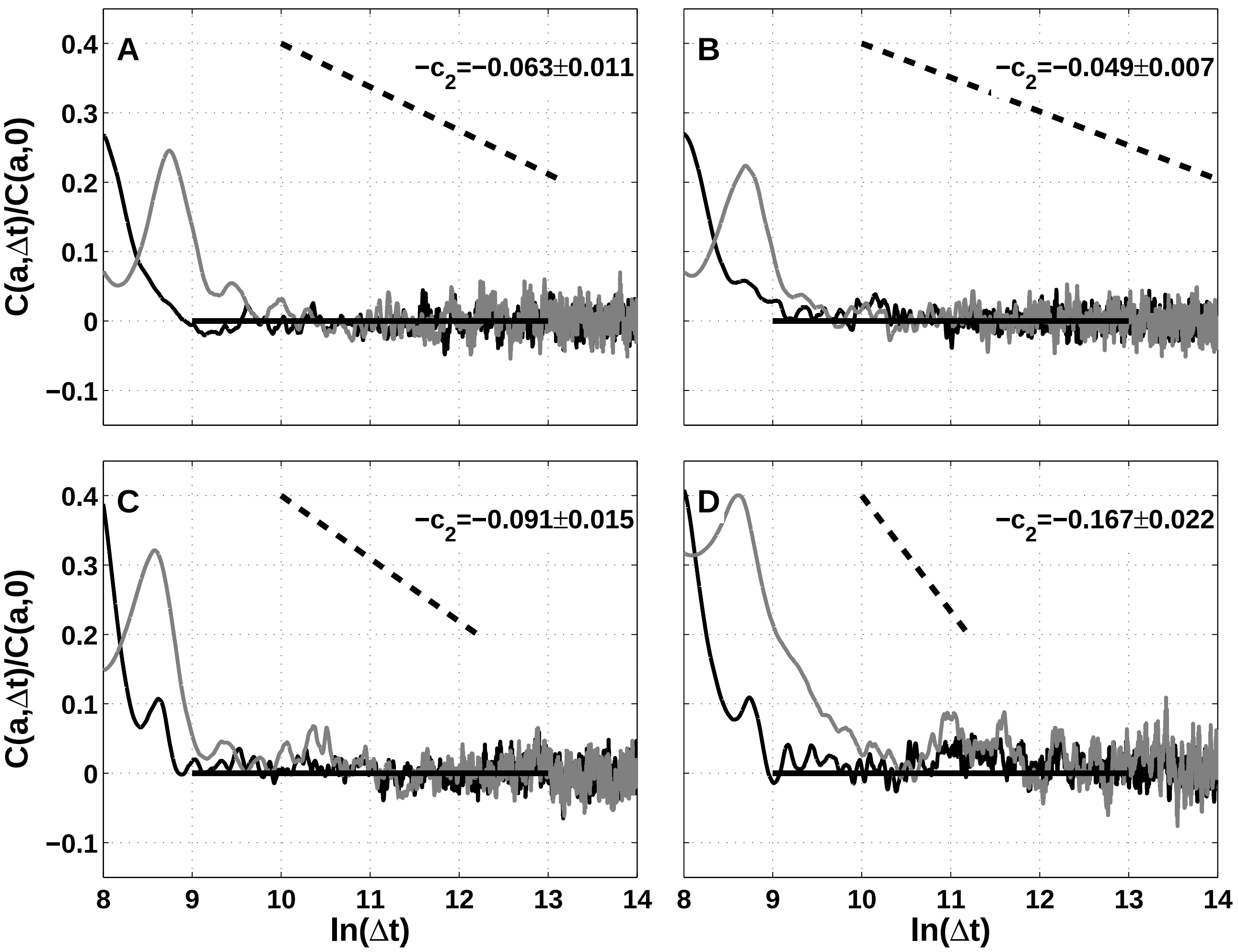

Figure S18: Two-point magnitude analysis of local impulse energy time-series recorded along the CS vein. Two-point correlation function $C(a,\Delta t)/C(a,0)$ *vs* $\ln(\Delta t)$ (Eq. (13)) for local impulse energy $E(t)$ computed with the analyzing wavelet $g^{(2)}$. The two curves correspond to scales $a = 2^9$ (black) and 2^{10} (grey) within the scaling range. (A) Pt1, (B) Pt2, (C) Pt3, and (D) Pt5.
